# Stimulation of Lignan Production in *Schisandra rubriflora* In Vitro Cultures by Elicitation

**DOI:** 10.3390/molecules27196681

**Published:** 2022-10-07

**Authors:** Agnieszka Szopa, Michał Dziurka, Paweł Kubica, Karolina Jafernik, Oliwia Siomak, Halina Ekiert

**Affiliations:** 1 Chair and Department of Pharmaceutical Botany, Faculty of Pharmacy, Medical College, Jagiellonian University, ul. Medyczna 9, 30-688 Kraków, Poland; 2 The Franciszek Górski Institute of Plant Physiology, Polish Academy of Sciences, ul. Niezapominajek 21, 30-239 Kraków, Poland

**Keywords:** *Schisandra rubriflora*, *Schisandra chinensis*, red-flowered Chinese magnolia vine, Chinese magnolia vine, sex lines, lignans, biotic and abiotic elicitors, bioreactors, temporary immersion systems

## Abstract

The study investigated the effect of elicitation with: chitosan (CH) (200 mg/L), yeast extract (YeE) (3000 mg/L), ethephon (ETH) (25 µM/L), and methyl jasmonate (MeJA) (50 µM/L), on lignan accumulation in agitated and bioreactor (Plantform temporary immersion systems) microshoot cultures of female (F) and male (M) *Schisandra rubriflora* Rehd. et Wils. (Schisandraceae) lines. The elicitors were supplemented on the 10th day of culture. Biomasses were collected at 24 h and 48 h, and 4, 6, and 8 days after the addition of each elicitor. The 24 compounds from the dibenzocyclooctadiene, aryltetralin, dibenzylbutane, and tetrahydrofuran lignans and neolignans were determined qualitatively and quantitatively in biomass extracts using the UHPLC–MS/MS method. The highest total contents [mg/100 g DW] of lignans were: for CH-95.00 (F, day 6) and 323.30 (M, 48 h); for YeE 104.30 (F, day 8) and 353.17 (M, day 4); for ETH 124.50 (F, 48 h) and 334.90 (M, day 4); and for MeJA 89.70 (F, 48 h) and 368.50 (M, 24 h). In the biomass extracts of M cultures grown in bioreactors, the highest total lignan content was obtained after MeJA elicitation (153.20 mg/100 g DW). The maximum total lignan contents in the biomass extracts from agitated and bioreactor cultures were 3.29 and 1.13 times higher, respectively, than in the extracts from the non-elicited cultures. The poor understanding of the chemical composition and the lack of studies in the field of plant biotechnology of *S. rubriflora* emphasize the innovativeness of the research.

## 1. Introduction

The Schisandraceae family includes twenty-seven species of the genus *Schisandra*. In modern phytotherapy, only one of them is most often used—*Schisandra chinensis* (Turcz.) Baill.—Chinese magnolia vine. Knowledge of the medicinal and cosmetic properties and alimentary value of the raw material—*Schisandrae chinensis fructus*, came from traditional Chinese medicine (TCM) [1,2]. The European monograph, the raw material appeared for the first time in 2008 in the European Pharmacopoeia 6th [3]. The raw material is also known in North America [4]. It also has a monograph in the International Pharmacopoeia published by WHO [5]. *S. chinensis* fruit extract shows inter alia, hepatoprotective, adaptogenic, anti-inflammatory, antibacterial and antioxidant activities [2,6,7]. Knowledge about the phytochemical composition and medicinal or alimentary properties of other species of the *Schisandra* genus, compared to *S. chinensis*, is very poor [8].

The subject of this study is the endemic, dioecious species of the genus *Schisandra* that occurs naturally only in the western part of the Sichuan province (south-western part of China)-*Schisandra rubriflora* Rehd. et Wils. (*Schisandra chinensis*). It’s a species known in TCM; mainly used as a tonic and sedative. It is also recommended in the treatment of hepatitis, chronic gastroenteritis, and neurasthenia [8,9,10].

The dioecious and low resistance to frost of *S. rubriflora* make the species mainly cultivated as ornamental plants in some regions of world (incl. Europe and north America) [8].

*S. rubriflora* does not have an official pharmacopoeial monograph. In the scientific literature, there are individual articles by teams from China on the development of the chemical composition and research on the biological activity of *S. rubriflora* leaves, shoots and fruits. Phytochemical studies focus on the main group of metabolites characteristic of the *Schisandra* genus-dibenzocyclooctadiene lignans [11,12,13,14]. The study of therapeutic properties concerns the antiviral activity (anti-HIV-1) [15], and the influence on the level of glutamine-pyruvate transaminase (GPT) [16].

Our previous research focused on *S. rubriflora* phytochemical and biotechnological research. The phytochemical composition of leaves, stems and fruits was developed in terms of polyphenolic compounds-phenolic acids and flavonoids, as well as a lignan profile taking into account the sex of individuals [11,17,18]. Moreover, the biological activity of the extracts was determined as anti-inflammatory and antioxidant [17,18]. In addition, the initiation and optimization with regard to optimal plant growth regulators’ (PGRs) composition and the duration of the growth period of *S. rubriflora* in vitro microshoot agar cultures were performed [17].

As part of this study, the cultures were adapted to agitated mode of growth as well as to maintain them in specific Plantform bioreactors (Swedish-made temporary immersion systems). The subject of the research was the further biotechnological optimization of the conditions for *S. rubriflora* in vitro cultivation based on testing various elicitation methods. Nowadays elicitation is a very useful tool of biotechnological studies aimed at boosting the production of secondary metabolites under in vitro conditions.

Elicitation is one of the most effective and currently widely used biotechnological tools for increased biosynthesis and accumulation of secondary metabolites of high biological value in in vitro cultures of various plant species [19,20,21].

Through this study, the biotic-chitosan (CH) and yeast extract (YeE), as well as abiotic elicitors methyl jasmonate (JaMe) and ethephon (ETH) were tested. The elicitors were applied on the 10th day of cultivation, and the harvesting of culture biomass was after 24 h, 48 h, and 4, 6, and 8 days.

The aim of the work was to intensify the production of lignans in the biomass of *S. rubriflora* microshoot cultures. The research included the female (F) and male (M) culture lines. The analyzes of the UHPLC–MS/MS method included four groups of lignans: dibenzocyclooctadiene, as well as aryltetralin, dibenzylbutane, tetrahydrofuran and dihydrobenzofuran neolignans.

## 2. Results and Discussion

### 2.1. Influence of Elicitation in Agitated Cultures

#### 2.1.1. The Biomass Appearance after Elicitation

**Figure 1:**
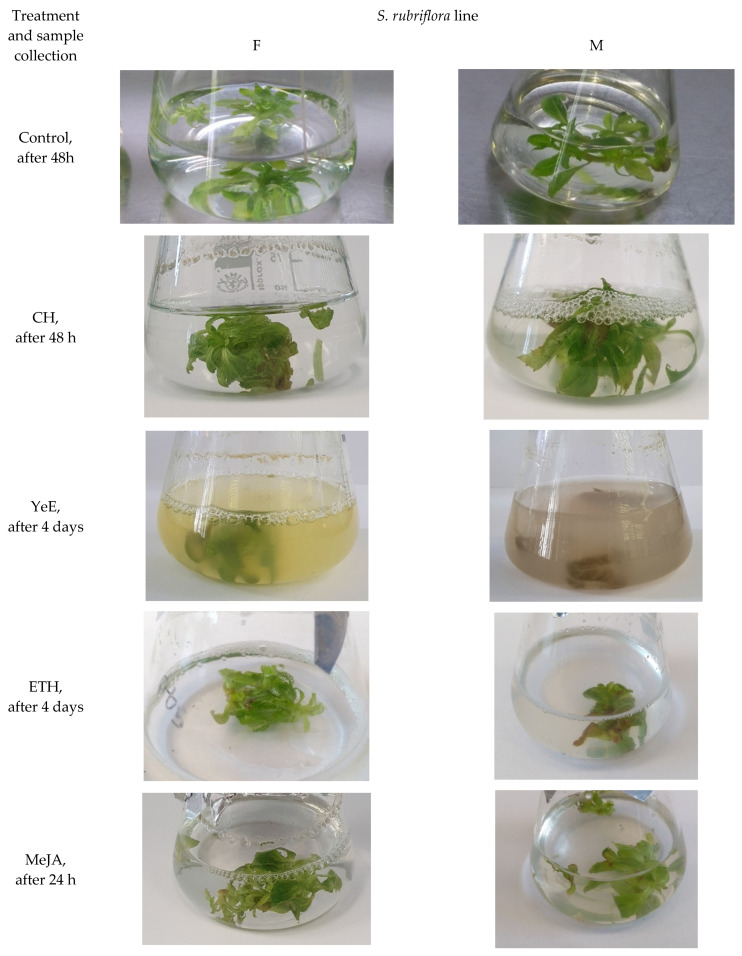
Examples of *S. rubriflora* agitated microshoot F and M lines appearance–control and after elicitor treatment.

Control, non-elicited cultures of *S. rubriflora* lines F and M showed good viability and a light green color after all tested growth periods (Figure 1). Progressive browning of biomass was observed in *S. rubriflora* cultures of lines F and M after supplementation with CH, ETH and MeJA substrates 6 days after the addition of elicitors. After YeE supplementation, progressive turbidity of the medium and gradual browning of microshoots were immediately observed (Figure 1). Similar effects of elicitors were observed in experiments conducted on shoot cultures of, e.g., *S. chinensis* [22], *Nasturtium officinale* [23] and *Eryngium planum* [24].

#### 2.1.2. Influence of Elicitation on Biomass Growth

In this study, the effect of the application of all elicitors on the biomass growth of agitated microshoot cultures of F and M lines of *S. rubriflora* was found. The highest biomass growth for line M was found 24 h after the addition of YeE. It caused the highest biomass gain, 2.06 times higher than the control. For line F, the highest biomass increase was found at 8 days after MeJA addition. It caused the highest biomass gain, 2.39 times higher biomass gain compared to the control (Table 1).

At 24 h after the addition of the elicitor, the biomass gains of elicited cultures, except for MeJA elicitation for lines M, were higher than those of the control cultures. The highest biomass gain of lines M at 24 h was recorded for YeE elicitation (max. Gi = 41.76), it was 2.06 times higher compared to the control. At 24 h after addition, elicitors did not affect the biomass gains of lines F except for YeE elicitation (max. Gi = 23.35). This gain was 1.28 times higher compared to the control (Table 1).

At 48 h after the addition of the elicitor, higher biomass gains were recorded for lines M except for those elicited with ETH and MeJA. The highest biomass gain of lines M was found for CH elicitation at 48 h (max. Gi = 37.57), it was 1.43 times higher compared to the control. The elicitors at 48 h after their addition also affected the biomass gain of the female lines except for MeJA elicitation. A high value of Gi coefficient was also found after ETH elicitation (max. Gi = 27.47). This gain was 1.94 times higher compared to the control (Table 1).

Supplementation with elicitors had a negative effect on the biomass gains of the experimental cultures harvested after 4 days. Only after CH elicitation for the biomass of line F, the Gi value (26.67) was 1.07 times higher compared to the control (Table 1).

For microshoots of agitated cultures of lines M 6 days after the addition of elicitors, only CH and MeJA showed a slight increase in Gi values compared to the control. The highest increase in biomass of line M was found after CH elicitation (max. Gi = 52.63), it was 1.16 times higher compared to the control (Table 1). For line F, only two elicitors, CH and YeE, affected biomass growth after 6 days of addition. The highest Gi value was found after YeE elicitation (max. Gi = 30.00). This increase was 1.32 times higher compared to the control (Table 1).

After 8 days of elicitation application for line M cultures, only CH and MeJA were found to have a higher Gi value compared to the control cultures. The highest biomass gain for lines M was found for MeJA elicitation (max. Gi = 69.26), this was 1.5 times higher than for the control. On the other hand, for line F, the highest Gi value was found after MeJA elicitation (Gi = 42.99). This gain was 2.39 times higher compared to the control (Table 1).

The notable Gi values in the same time points are diverse. That is caused by the type of elicitor and the duration time of elicitor treatment. The elicitor addition often caused a decrease in in vitro culture biomass growth while the production of secondary metabolites increase. Such a fact is well known in plant biotechnology studies and has been described before, e.g., after CH elicitation in *Hypericum perforatum* root cultures [25], MeJA elicitation in *Fagonia indica* adventitious root cultures [26], YeE elicitation in *Panax ginseng* cell culture [27], or *Aspergillus flavus* fungus elicitation in *Catharanthus roseus* callus cultures [28].

Analysis of the literature allows a direct comparison of data on the effect of elicitation with two elicitors: YeE and CH on the value of the Gi index for in vitro cultures of *S. chinensis* species, and the experiment carried out in the present work. The highest recorded Gi index values for YeE elicitation of *S. chinensis* species were observed after YeE addition at a concentration of 1000 mg/L. The Gi value ranged from 33.37 to 42.89, where the value for the control sample was 40.74. The other, higher concentrations of YeE, 3000 mg/L and 5000 mg/L, caused a decrease in biomass growth especially when YeE was supplemented on the first day of culture [22]. In the course of our experiment, a decrease in the biomass growth of in vitro cultures of *S. rubriflora* was found 4 days after the addition of YeE at a concentration of 3000 mg/L. For line M it was also observable on day 6 and day 8 of culture. Line M of *S. rubriflora* was found to be much more sensitive to YeE compared to line F. Elicitation of *S. chinensis* with CH at concentrations in the range of 25–200 mg/L did not adversely affect microshoots growth. The Gi index was comparable to control cultures. The highest Gi value was found for CH supplementation at concentrations of 50 and 100 mg/L on the first day of culture. It was 57.39 and 56.87, respectively; where the Gi value for the control sample was 40.00 [22]. In the course of our experiment for in vitro cultures of *S. rubriflora*, the increase in CH elicited biomass for cultures of line F occurred only at 48 h after elicitor addition, it was the highest at day 8 after elicitor addition, while cultures of line M showed the highest increase at 24 h after elicitor addition (Table 1).

#### 2.1.3. The Influence of Elicitation on Lignan Production

The study proved the significant effect of the applied elicitation on the accumulation of lignans in cultures of lines F and M of *S. rubriflora*. In biomass extracts, 22 compounds from four groups of lignans were qualitatively and quantitatively determined: dibenzocyclooctadiene lignans (schisantherin A and B, schisandrin, schisandrin C, gomisin A, D, G, J, N, O, 6-O-benzoylgomisin O, schisandrin A, rubrisandrin A, epigomisin O, schisanhenol, interiotherin C, angeloylgomisin H and O), aryltetralin lignans (wulignan A1), dibenzylbutane lignans (pregomisin, mesodihydroguaiaretic acid), and tetrahydrofuran lignans (fragransin A2). In addition, 2 compounds from the dihydrobenzofuran group of neolignans (licarin A and B) were also found in the analyzed extracts.

The study confirmed the effect of the elicitation schemes used on the accumulation of metabolites in the microshoot culture biomass extracts analyzed, while only trace amounts were found in the culture media (<5 mg/L).

Detailed results of quantitative analyses in the control sample and depending on the elicitation scheme used and on the time of tissue harvesting are presented in Supplementary Data (Tables S1–S5).

The highest obtained contents of the analyzed compounds in the course of the whole experiment were as follows: wulignan A1 (max. 0.36 mg/100 g DW; CH, line F, 6 days, 2.37 times higher compared to the control), rubrisandrin A (max. 0.19 mg/100 g DW; YeE; line M, 6 days, 3.82 times higher compared to the control), interiotherin C (max. 0.36 mg/100 g DW; CH; line F, 6 days, 7.16 times higher compared to the control), schisandrin (max. 71,98 mg/100 g DW; MeJA, line M, 24 h, 3.13 times higher compared to the control), gomisin D (max. 28.80 mg/100 g DW; MeJA, line M, 24 h, 4.44 times higher compared to the control), gomisin J (max. 15.71 mg/100 g DW; MeJA, line M, 24 h, 4.78 times higher compared to the control), gomisin A (max. 91.53 mg/100 g DW; MeJA, line M, 24 h, 3.31 times higher compared to the control), gomisin G (max. 6.93 mg/100 g DW; MeJA, line M, 24 h, 3.91 times higher compared to the control), licarin B (max. 0.41 mg/100 g DW, ETH, line F, 48 h, 3.33 times higher compared to the control), epigomisin O (max. 1.54 mg/100 g DW; ETH, line F, 48 h, 2.40 times higher compared to the control), gomisin O (max. 4.80 mg/100 g DW; MeJA, line F, 48 h, 1.38 times higher compared to the control), mesodihydroguaiaretic acid (max. 0.23 mg/100 g DW; MeJA, line M, 8 days, 4.62 times higher compared to the control), schisantherin A (max. 5.42 mg/100 g DW; ETH, line F, 48 h, 1.99 times higher compared to the control), schisantherin B (max. 16.66 mg/100 g DW; MeJA, line M, 24 h, 5.03 times higher compared to the control), licarin A (max. 37.54 mg/100 g DW; YeE, line F, 8 days, 3.23 times higher compared to the control), schisanhenol (max. 10.11 mg/100 g DW; ETH, line F, 48 h, 1.79 times higher compared to the control), deoxyschisandrin (max. 94.86 mg/100 g DW; YeE, line M, 4 days, 1.65 times higher compared to the control), gomisin N (max. 28.07 mg/100 g DW; MeJA, line M, 24 h, 4.14 times higher compared to the control), 6-O-benzoylgomisin O (max. 1.40 mg/100 g DW; ETH, line F, 48 h, 2.64 times higher compared to the control), and schisandrin C (max. 5.24 mg/100 g DW; YeE, line M, 24 h, 3.64 times higher compared to the control) (Tables S1–S5 and Table 2, Table 3, Table 4, Table 5 and Table 6).

Total content of lignans was highest for line M elicited with MeJA, and was 368.50 mg/100 g DW, 3.29 times higher than in the control sample (Tables S1–S5 and Table 2, Table 3, Table 4, Table 5 and Table 6).

The obtained results were compared with elicitation in in vitro cultures of well-established in phytotherapy *S. chinensis* which affected the production of dibenzocyclooctadiene lignans [22]. Cultures of *S. chinensis* were maintained on MS medium containing 3 mg/L BA and 1 mg/L NAA. Cultures were treated with elicitors at 10 and 20 days of culture. The elicitors tested were cadmium chloride (CdCl_2_), YeE, CH, MeJA and the permeabilizing agent dimethyl sulphoxide (DMSO). The duration of culture was 30 days. In the biomass extracts of the experimental cultures, the content of dibenzocyclooctadiene lignans was determined by DAD-HPLC. The total content of lignans obtained in cultures elicited with CdCl_2_ was twice as high as in non-elicited control samples. The content of secondary metabolites after the use of this elicitor was about 730 mg/100 g DW. Elicitation with CH increased lignan production by 1.35 times (500 mg/100 g DW). The use of YeE led to a 1.8-fold increase in the content of the tested compounds. The identified lignans differed from those determined in in vitro cultures of *S. rubriflora* species during the present experiment. In the *S. chinensis* species, only lignans from the dibenzocyclooctadiene lignan group were determined: schisandrin, gomisin A, gomisin G, schisantherin A, schisantherin B, Schisanhenol, deoxyschizandrin, γ-schisandrin, schisandrin C, angeloylgomisin H and Q, schisandrin B, benzoylgomisin P and schisantherin D. Only the effect of elicitation on the compounds found in both species, i.e., dibenzocyclooctadiene lignans, was evaluated comparatively in this study (compounds from the other labeled lignan groups in *S. rubriflora* were not considered): schisandrin, gomisin A, G, schisantherin A, B, schisanhenol, deoxychizandrin and the total content of these compounds (Table 7). Schisandrin, gomisin A and G, and schisantherin B showed higher content multiplicity relative to the control due to elicitation in *S. rubriflora* species (3.13, 3.31, 3.91, 5.03 times higher for MeJA, respectively, 50 µM, 24 h,) than in *S. chinensis* species (2.28, 2.80 times more for CdCl_2_, respectively, 1000 µM, 10 days, 3.03 times more for YeE, 3000 mg/L, day 10; 2.61 times more for YeE, 1000 mg/L, 20 days). The content of these compounds was lower in elicited cultures of *S. rubriflora* species. Schisantherin A, schisanhenol, deoxyschizandrin showed lower multiplicity of content relative to control upon elicitation in *S. rubriflora* species (1.99, 1.79 times more, respectively, ETH, 25 µM, 48 h, 1.65 times more, YeE, 3000 mg/L, 4 days) in cultures of *S. rubriflora* species than in those for *S. chinensis* (3.11 times more, YeE, 3000 mg/L, day 0; 4.44 times more, CdCl2, 1000 µM, 10 days; 1.95 times more, YeE, 5000 mg/L, days 10). The contents of schisantherin A and schisanhenol were lower in *S. rubriflora* cultures than in S. *chinensis*. The only compound that showed a higher content in *S. rubriflora* cultures was deoxyschizandrin (max. 94.86 mg/100 g DW). The total content of lignans was higher in *S. chinensis* cultures (max. 730.60 mg/100 g DW) than in *S. rubriflora* (max. 368.50 mg/100 g DW), while the multiplicity relative to the control was higher in *S. rubriflora* cultures (3.29 times higher) (Table 7).

Studies on the effect of elicitation on the production of specific compounds from the lignan group are a rather difficult subject of research, and that is probably why they are not a frequent object of study. Studies on a popular compound with anticancer activity-podophyllotoxin are of greatest interest. Kasparova et al. conducted a study on the effect of MeJA elicitation on podophyllotoxin accumulation in suspension cultures of *Juniperus virginiana*. Cultures were elicited on day 14 of culture and harvested at 6, 24, 48 and 168 h after the addition of the elicitor. The highest maximum podophyllotoxin content was determined at 168 h after the addition of 5 mmol/L MeJA (max. 0.68 mg/g DW) and was about 2.83 times higher than the control sample [29]. Anbazhagan et al. conducted a study on the effect of MeJA elicitation on podophyllotoxin accumulation in suspension and adventitious root cultures of *Podophyllum peltatum* species. Day “0” (inoculation) was the day of addition of the elicitor 20 μM MeJA. Cultures were harvested 5 weeks after elicitor addition. Podophyllotoxin content in suspension cultures was 4.1 times higher than in the control samples (max. 0.3625 mg/g DW), and in adventitious root cultures was 1.62 times higher than in the control samples (max. 0.588 mg/g DW) [30]. Bhattacharyya et al. conducted a study on the effect of MeJA elicitor on podophyllotoxin accumulation in *Podophyllum hexandrum* species. Elicitor; 100 µM MeJA, was added on day 3 of culture. Cultures were harvested after 9 days. Podophyllotoxin content was 80 µg/g DW and was 8 times higher than in the control samples [31].

Sasheva et al. conducted a study on the effect of MeJA elicitation on podophyllotoxin accumulation in *Linum thracicum* species. MeJA was used at concentrations of 50 and 100 µM and added on day 7 of culture. Cultures were harvested 24 h and 72 h after the addition of the elicitor. Cultures were grown on MS media differing in sucrose content. The highest content of podophyllotoxin was 1.6 mg/g DW, which was 1.14 times higher than in the control sample and was obtained in biomass cultured on medium with 20 g/L sucrose. The researchers found no significant effect of elicitation on the production of podophyllotoxin [32].

Waqar et al. studied the effect of CH elicitation on the accumulation of specific lignans and neolignans: secoisolariciresinol diglucoside, lariciresinol diglucoside, dehydrodiconiferyl alcohol glucoside and guaiacylglycerol-β-coniferyl alcohol ether glucoside in suspension cultures of *Linum usitatissimum*. Cultures were elicited with CH at a concentration of 10 mg/L on day 10 of culture. The duration of culture was 30 days. Material was harvested at 8, 24 and 48h. Maximum enhancements of 7.3-fold (28 mg/g DW) occurred for lariciresinol diglucoside, 3.5-fold (58.85 mg/g DW) in dehydrodiconiferyl alcohol glucoside and while the least enhancement of 2-fold (18.42 mg/g DW) for secoisolariciresinol diglucoside was observed in CH treated cell cultures than to controls [33]. Nadeem et al. also studied the effect of elicitation, but with a different elicitor, YeE, on the accumulation of lignans and neolignans in suspension cultures of *L. usitatissinum* species. YeE was added to the medium on day “0” (inoculation) at concentrations of 10, 50, 100, 200, 500 and 1000 mg/L. YeE at a concentration of 200 mg/L caused the highest increase in the accumulation of the compounds: secoisolariciresinol diglucoside (3.36-fold, max. 10.1 mg/g DW), lariciresinol diglucoside (1.3-fold, 11.0 mg/g DW) and dehydrodiconiferyl alcohol glucoside (4.26-fold, max. 21.3 mg/g DW) [34].

Wawrosch et al. studied the effects of elicitation with AgNO_3_, MeJA and YeE on the accumulation of specific furan-type lignans: leoligin and 5-methoxy-leoligin in hairy root cultures of *Leontopodium nivale*. Elicitors were added to 3-week-old cultures at concentrations of: 15, 30 and 60 µM AgNO_3_, 50, 100, 200, 300 µM MeJA and 1, 2, 5 g/L YeE. The duration of culture was 4 weeks. The highest content of leoligin was recorded after elicitation with 100 µM MeJA (max. 0.05 in %), it was 8.33 times higher than that of the control sample. The content of 5-methoxy-leoligin was highest after supplementation with 15 µM AgNO_3_ (max. 0.026 in %), and was 6.5 times higher than that of the control sample [35].

Schmitt and Petersen examined the effect of MeJA elicitation on the accumulation of tetrahydrofuran lignans, pinoresinol and matairesinol, in *Forsythia* × *intermedia* suspension cultures. MeJA was added on day “0” at a concentration of 100 µM. Cultures were harvested every other day from the addition of the elicitor. Pinoresinol content increased 3-fold (max. 0.8 mg/g DW) and matairesinol content increased 7-fold (max. 2.7 mg/g DW) relative to the control sample [36].

Sanchez-Sampedro et al. conducted an experiment proving the effect of elicitation with YeE, SA, CH and chitin on the synthesis of flavonolignan: silymarin in suspension cultures of *Silybum marianum*. SA, CH and chitin did not stimulate silymarin production even at higher concentrations. YeE caused intense browning and significant loss of cell viability after 48 h (at concentrations of 100 and 200 μg/mL). A slight increase in silymarin content was observed following YeE supplementation. MeJA at a concentration of 10 µM/mL was ineffective, but at a concentration of 100 µM/mL caused significant accumulation of silymarin in cells. MeJA alone or in combination with YeE gave the best results. Three-day cultures were treated for 48 h with 50 g/mL YeE, 100 μM MeJA or both elicitors simultaneously. Silymarin content for control samples was max 2.01 mg/g DW. The combination of MeJA and YeE yielded nearly 600% higher accumulation of silymarin in biomass [37].

### 2.2. Influence of Elicitation in Cultures Maintained in Plant form TIS

#### 2.2.1. The Biomass Appearance and Growth after Elicitation

**Figure 2:**
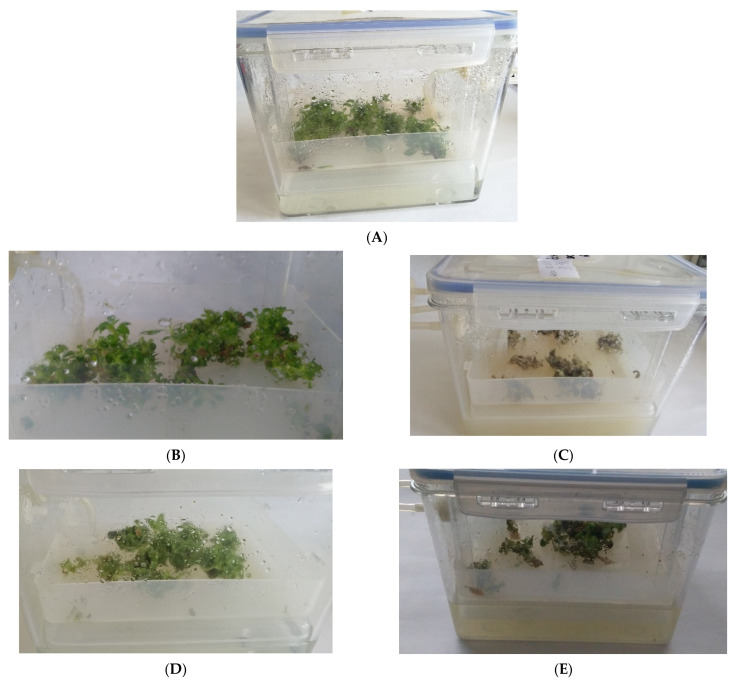
Biomass appearance of control and elicited in vitro cultures of *S. rubriflora* line M grown in Plantform bioreactors: (**A**)—control, (**B**)—CH, (**C**)—YeE, (**D**)—ETH, (**E**)—MeJA.

Experimental cultures of *S. rubriflora* line M grown in Plantform bioreactors were characterized by good growth and light green color of microshoots (Figure 2). The elicitor concentrations used for the bioreactor experiments and the days of biomass harvesting from its supplementation were selected based on the best results obtained in agitated culture experiments. The collection of samples supplemented with each elicitor was as follows: MeJA at 24 h, CH at 48 h, YeE at day 4, ETH at day 4, control at day 4. The effect of the elicitors used on the appearance of microshoots was observed only in the case of YeE and MeJA elicitation, where the tissue was slightly browned after elicitor supplementation (Figure 2).

**Figure 3:**
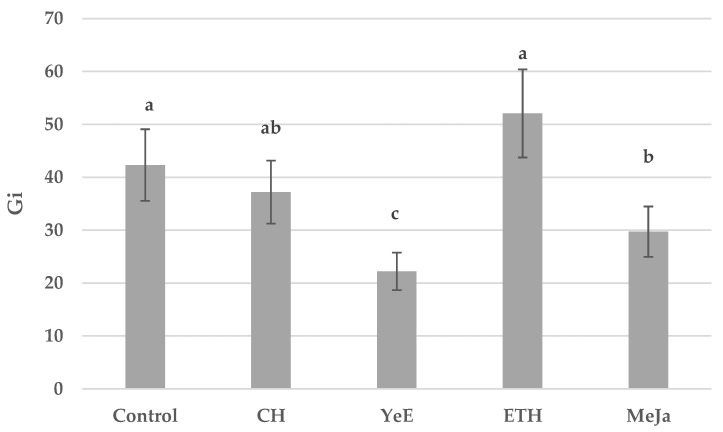
Biomass gains of control and elicited in vitro cultures of *S. rubriflora* grown in Plantform bioreactors. Data expressed as the mean value ± SD (*n* = 3). Different superscript letters (a–c) within a row indicate significant differences between means (Duncan’s multiple range test; *p* < 0.05).

Biomass gains of the control sample in Plantform bioreactors were Gi = 42.31. Dry biomass gains for elicited samples were as follows: CH; Gi = 37.21, YeE; Gi = 22.22, ETH; Gi = 52.07, and MeJA; Gi = 29.72 (Figure 3).

#### 2.2.2. The Influence of Elicitation on Lignan Production

The presence of compounds, the same as in extracts from agitated cultures, was confirmed qualitatively in the biomass extracts of control and elicited bioreactor cultures: dibenzocyclooctadiene lignans (schisantherin A and B, schisandrin, schisandrin C, gomisin A, D, G, J, N, O, 6-O-benzoylgomisin O, schisandrin A, rubrisandrin A, epigomisin O, schisanhenol, interiotherin C, angeloylgomisin H and O), aryltetralin lignans (wulignan A1), dibenzylbutane lignans (pregomisin, mesodihydroguaiaretic acid) and tetrahydrofuran lignan (fragransin A2), and compounds from the group of dihydrobenzofuran neolignans (licarin A and B).

The contents of individual compounds ranged from trace amounts <0.05 mg/100 g DW to 41.01 mg/100 g DW (gomisin A, MeJA, 24 h). The highest contents of the individual compounds analyzed in the course of the entire experiment were as follows: schisandrin (max. 37.60 mg/100 g DW; MeJA, 1.25 times higher than the control sample), gomisin A (max. 41.01 mg/100 g DW; MeJA, 1.03 times higher than the control sample), and deoxyschisandrin (max. 35.00 mg/100 g DW; MeJA, 1.06 times higher than the control sample) (Table 8).

Total lignan contents ranged from 114.80 mg/100 g DW (ETH, 4 days) to 153.20 mg/100 g DW (MeJA, 24h) (Table 8). The maximum total lignan content obtained was 1.13 times higher than for the control sample.

Trace amounts of lignans were found in culture media (<0.05 mg/100 g DW).

In general, for cultures grown in Plantform bioreactors, the most effective elicitor for which the highest individual and total lignan contents were obtained, was MeJA.

The accumulation of active compounds in plant cultures grown in bioreactors is less frequently studied due to the fact that for these experiments a much larger amount of plant tissue is required to initiate the experiment, as well as the availability of special bioreactor-like structures being limited [38]. Studies on the effect of YeE elicitation on dibenzocyclooctadiene lignan production in *S. chinensis* microshoot cultures maintained in Plantform bioreactors have been studied by us before [22]. Results proved that the supplementation with 1000 mg/L YeE on the 20th day of the growth cycle was the optimal. Through this elicitation scheme the total content of the estimated dibenzocyclooctadiene lignans was equal to 831.60 mg/100 g DW. The dominant dibenzocyclooctadiene lignans were schisandrin-186.8 mg/100 g DW, angeoyl/tigloyl-gomisin Q–183.4 mg/100 g DW and deoxyschisandrin–100.00 mg/100 g DW. In the cultures of *S. rubriflora* we tested, maintained in Plantform bioreactors, different lignans were proven to be dominant and their amounts were of lower order (Table 8).

The effect of elicitation treatments on the production of other lignan groups in the biomass of plant in vitro cultures grown in bioreactors is a new and little-exploited research direction [38]. Dougué Kentsop et al. recently tested production of arylnaphthalene lignan-justicidin B, in *L. lewisii* adventitious and hairy-roots cultures maintained in the stirred tank bioreactor. Both of the culture types were grown in the bioreactor for 3 weeks and then elicited with 100 μM MeJA and grown for a further one week. The justicidin B content in both cultures after treatment with MeJA doubled in comparison to the control and was to equal 99.2 and 132.6 mg/g DW, respectively [39].

### 2.3. Biotechnological Evaluation of Elicitation Results

The compounds obtained in the course of the experiment were analyzed, and their highest contents were extracted taking into account the elicitation conditions. The collected data were compared in relation to the multiplicity of the content increase in relation to the control and compared with the results of the analysis of the material obtained from the parent plants (Table 9).

For a number of compounds: rubrisandrin A, interiotherin C, schisandrin, gomisin D, J, N and A, schisantherin A, licarin A, and schisandrin C, their contents in extracts from in vitro cultures were higher than in extracts from fruits of parent plant. This is important, as the fruit is widely recognized as *Schisandra*’s raw material. Our study also proved higher values for individual compounds in extracts from in vitro cultures compared to the extracts from leaves and stems of parent plant material (Table 9). In this context, the results obtained have a potential applied nature.

Total lignan content was highest for the line M elicited with MeJA and was 368.50 mg/100 g DW and was 3.29 times higher than that of the control-non-elicited cultures. The content was 1.3 times higher than in the shoots, but 2.6-times lower than in the leaves and 2.1-times lower than in the fruit of the parent plant (Table 9).

## 3. Materials and Methods

### 3.1. Plant Material and Microshoot Culture Initiation

Plant material for in vitro culture initiation was obtained as part of cooperation with Clematis–Źródło Dobrych Pnączy (Pruszków, Poland) [40]. Moreover, the fruits, leaves and stems of the parent plant material were obtained from this arboretum. Plant species were identified by Dr. Szczepan Marczyński and Dr. Agnieszka Szopa. For these purposes the leaf buds of about 10 years old female (F) (100 individuals) and male (M) (50 individuals) *S. rubriflora* (Franch.) Rehd. et Wils specimens were collected in May 2018. Leaf buds were defatted with 70% ethanol (30 s) and then sterilized for 7 min with 0.1% HgCl_2_ (mercuric chloride II). Sterile buds were rinsed three times with sterile redistilled water and transferred to agar medium according to Murashige and Skoog (1962) (MS) [41] containing 1 mg/L BA (6-benzyladenine) and 0.5 mg/L NAA (1-naphtaleneacetic acid).

Agar (Duchefa Biochemie, 7.2 g/L) microshoot cultures of the male (M) and female (F) lines of *S. rubriflora* were run in Magenta TM B-cap dishes (product no. V8630-Sigma-Aldrich^®^, diameter: 60 mm, height: 70 mm, capacity: 100 mL), which each contained 30 mL of MS medium (pH 5.7–5.8). These microshoots were used to initiate experimental cultures.

### 3.2. Experimental Agitated Cultures

Experimental microshoot cultures were obtained by passaging 0.75 g of inoculum (initial fresh weight of microshoots) per 1 vessel (150 mL Erlenmayer flasks) containing 50 mL of standard liquid MS medium (without agar) supplemented with 1 mg/L BA and 1 mg/L IBA (indole-3-acetic acid). Agitated cultures were grown under constant artificial light (light-emitting diode (LED) white light, 90 ± 2 μmol m^−2^ s^−1^) at 24 ± 2 °C for 10 days. The addition of the individual elicitor (5 flasks per series; 3 culture series were carried out) followed on day 10 (Figure 1). Biomass harvesting occurred at: 24 h, 48 h, and the 4th day, 6th day, and 8th day after the addition of the elicitor. At the same time, control samples were run and harvested at appropriate intervals (not elicitor treated).

The culture media contained the following concentrations of elicitors: 200 mg/L of chitosan (CH; Sigma-Aldrich, St. Louis, MO, USA), 3000 mg/L of yeast extract (YeE; Sigma-Aldrich, St. Louis, MO, USA), 25 µM/L of ethephon (ETH; Sigma-Aldrich, St. Louis, MO, USA) and 50 µM/L of methyl jasmonate (MeJA; Sigma-Aldrich, St. Louis, MO, USA). The solutions of CH, ETH and MeJA were filter-sterilized using a 0.22 µm syringe filter (Millex^®^GP; Merck Millipore, Burlington, MA, USA), and the solution of YeE was autoclaved (at 121 °C, at a pressure of 0.1 MPa for 20 min) and added to the culture medium to obtain proper a concentration in the medium.

Stock solutions of each elicitor were prepared for the experiments. A concentrated solution of MeJA in which the concentration of MeJA was 0.00449 g/mL was prepared and added to flasks with experimental cultures to obtain a concentration of 50 µM/L. A concentrated solution of ETH in which the concentration of ETH was 3.6 mg/L was prepared and added to flasks with experimental cultures to obtain a concentration of 25 µM/L. A concentrated solution of CH in which the concentration of CH was 200 mg/L was prepared and added to flasks with experimental cultures to obtain a concentration of 50 µM/L. A concentrated solution of YeE was prepared according to the method of Peltonen et al. [42] in which the 30 mg/mL concentration of YeE was added to flasks with experimental cultures to obtain a concentration of 3 g/L.

### 3.3. Experimental Plantform TIS Cultures

Bioreactor cultures were conducted in commercial Plantform bioreactors-temporary immersion system-TIS (PlantForm company, Hjärup, Sweden). Experiments were conducted on microshoots of the M line of *S. rubriflora*. Nine grams of microshoots were inoculated per single bioreactor and 500 mL of MS medium with 1 mg/L BA and 1 mg/L IBA was used. Cultures were grown in constant artificial (light-emitting diode (LED) white light, 90 ± 2 μmol m^−2^ s^−1^), at 24 ± 2 °C, for 10 days. The flooding cycle of the bioreactors was set at 5 min every 90 min. The elicitor was added on the 10th day of culture. Elicitors were supplemented to the culture media at the same concentrations per volume of medium as were used in the agitated cultures. The choice of harvesting time for cultures conducted in bioreactors was dictated by the best results obtained at the agitated culture stage. Cultures were harvested depending on the elicitor used: MeJA after 24 h, CH after 48 h, YeE and ETH on day 4. There were 3 bioreactors for each elicitor. Three culture series were carried out.

### 3.4. Biomass Gains

Biomass gains of control samples, elicited samples and samples run in Plantform bioreactors for in vitro cultures of *S. rubriflora* lines F and M were measured. Biomass was separated from the culture medium and washed several times with redistilled water. To determine dry weight-DW (dry weight) gains, biomass was frozen at −20˚C, then freeze-dried (freeze dryer, Labconco Corporation, Kansas City, MO, USA) and weighed again. To determine biomass gains, growth index values for dry biomass-Gi (growth index) were calculated for F and M lines according to the formula [43]: $\mathrm{Gi}=\frac{\mathrm{Dwn}-\mathrm{Dwo}}{\mathrm{Dwn}}\times 100$, where Gi-growth index at time “n”; DWn-dry weight at time “n”; DWo-dry weight of inoculum.

### 3.5. Chromatographic Analysis of Lignans

Methanol extracts of biomass from the experimental cultures were prepared. Dry plant material was pulverized in a mixing ball mill (MM400, Retch, Haan, Germany) and dry biomass was weighed: 0.1 g from in vitro cultures of lines F and M. The material was then extracted with methanol (purity for HPLC analysis from: Merck)-2 mL. The extraction process was carried out twice in an ultrasonic bath (POLSONIC Palczynski Sp.J., Warsaw, Poland, Sonic 2 model) for 30 min. The obtained extracts were centrifuged for 5 min (4000 rpm) in a centrifuge (MPW Med. Instruments, model Centrifuge MPW-223E). The centrifuged extracts were filtered using sterilizing syringe strainers (Millex^®^GP, Millipore, pore diameter: 0.22 μm, Filter Unit) into appropriate vials for HPLC chromatographic analyses (Witko Sp.z.o.o.).

Lignans were determined by ultra-high performance liquid chromatography coupled to a tandem mass spectrometer (UHPLC–MS/MS) technique. The apparatus consisted of a UHPLC Infinity 1260 (Agilent, Wolbrom, Germany) and a 6410 MG/100 G DWQ LC/MS tandem quadrupole mass spectrometer (Agilent, Santa Clara, CA, USA). Samples in a volume of 2 µL were injected onto an analytical column (Kinetex^TM^ C18: 150 × 4.6 mm, 2.7 µm). The analytes were eluted in a gradient of 50% water in methanol (A) and 100% methanol (B) with the addition of 0.1% formic acid in both phases, from 20% to 65% solvent B for 22 min, at a mobile phase flow rate of 0.5 mL/min at 60 °C acc. to [11,18]. In addition, a DAD spectrophotometric detector was connected in the chromatographic system, and the tested compounds were monitored at a wavelength (λ) of 225 nm. For targeted profiling of lignans, a tandem quadrupole mass analyzer with electrospray ionization (ESI) was used in the positive atomic mass-to-charge ratio (m/z) ion monitoring mode. In order to obtain the greatest possible confidence in the identity of the compounds under study and the greatest possible sensitivity of the determination, the MRM (multiple reaction monitoring) technique was used, which involves selecting a single parent ion characteristic of the substance under study and then monitoring the progeny ions formed after collision with inert gas particles in a collision chamber. Standard lignan substances were purchased from ChemFaces Biochemical Co., Ltd. (Wuhan, China).

### 3.6. Statistical Analysis

Quantitative results are expressed in mg/100 g DW (dry weight) as the mean ± SD (standard deviation) of three replicates (*n* = 3). The influence of elicitor treatment was evaluated by one-way ANOVA. Differences between means were calculated using Duncan’s multiple range test (*p* < 0.05) using the statistical package STATISTICA 13.0 (Stat-Soft, Inc., Tulsa, OK, USA).

## 4. Conclusions

The present study is the first such complex biotechnological study aimed at wide elicitation protocol elaboration using biotic elicitors-chitosan (CH) and yeast extract (YeE), as well as abiotic elicitors-methyl jasmonate (JaMe) and ethephon (ETH), for boosting the production of unique compounds from lignan groups. These metabolites are characteristic of *S. rubriflora* endemic Chinese species. Through our study we proved, for the first time, possibilities for increasing their production in biomass cultures under in vitro conditions (in independence on environmental factors). Our study also described for the first time the influence of elicitation on lignan compounds’ production in the *S. rubriflora* Platform TIS bioreactors grown microshoot cultures. An important aspect of the research performed was also to compare the biosynthetic capabilities of the F and M lines, and thus the sexes on lignan production.

The results revealed a high competitiveness of *S. rubriflora* in vitro cultures in relation to soil grown plants. Our results showed new perspectives of potential in vitro cultures utilization as an alternative for a rare, hard-to-find plant material that is difficult to produce on an industrial scale.

## Figures and Tables

**Table 1:** Comparison of biomass gains (Gi ± SD) of F and M lines of *S. rubriflora* agitated cultures depending on harvesting time and the elicitor used. Data expressed as the mean value ± SD (*n* = 3). Different superscript letters (a–c) within a row indicate significant differences between means (Duncan’s multiple range test; *p* < 0.05).

Time of Harvesting	Control		Elicitor							
			CH		YeE		ETH		MeJA	
	F	M	F	M	F	M	F	M	F	M
**24 h**	18.30 b ± 8.20	20.24 b ± 1.43	12.93 bc ± 11.50	38.01 a ± 0.06	23.35 b ± 7.56	41.76 a ± 9.61	8.03 c ± 3.07	31.36 ab ± 0.37	9.88 c ± 12.31	6.04 c ± 6.88
**48 h**	14.19 ab ± 4.13	26.20 a ± 19.52	18.02 ab ± 4.57	37.57 a ± 17.60	15.78 ab ± 2.48	33.82 a ± 11.49	27.47 a ± 7.53	8.3 b ± 2.43	9.31 b ± 2.04	10.87 b ± 12.08
**4 days**	24.96 b ± 2.53	51.12 a ± 0.48	26.67 b ± 1.89	49.49 a ± 14.31	19.53 b ± 19.84	40.85 a ±13.32	14.29 c ± 7.45	20.52 b ± 0.60	12.56 c ± 11.59	18.07 b ± 15.34
**6 days**	22.76 b ± 7.99	45.37 a ± 9.24	26.69 b ± 1.97	52.63 a ± 6.59	30.00 b ± 7.07	34.07 ab ± 13.96	21.87 b ± 6.93	16.04 c ± 6.53	12.73 bc ± 15.43	49.3 a ± 9.54
**8 days**	17.95 b ± 23.23	45.98 b ± 11.89	32.27 b ± 10.97	57.95 a ± 6.25	31.51 b ± 10.49	27.37 b ± 16.78	24.95 b ± 9.61	27.77 b ± 8.64	42.99 b ± 7.78	69.26 a ± 1.64

**Table 2:** Accumulation of lignans (mg/100 g DW) in agitated cultures of *S. rubriflora* lines F and M 24 h after addition of elicitor. Data expressed as the mean value ± SD (*n* = 3). Different superscript letters (a–f) within a row indicate significant differences between means (Duncan’s multiple range test; *p* < 0.05).

Lignan	Control		Elicitor							
			CH		YeE		ETH		MeJA	
	F	M	F	M	F	M	F	M	F	M
Wulignan A1	0.10 a ± 0.01	0.02 b ± 0.002	0.11 a ± 0.003	0.05 b ± 0.01	0.05 b ± 0.002	traces	traces	traces	0.07 b ± 0.01	traces
Rubrisandrin A	traces *	0.06 ± 0.01	traces	traces	traces	traces	traces	traces	traces	traces
Interiotherin C	traces	traces	0.11 a ± 0.003	0.05 b ± 0.01	traces	traces	traces	traces	traces	traces
Schisandrin	13.90 d ± 0.82	23.00 c ± 2.46	11.64 d ± 0.36	34.26 b ± 3.79	11.09 d ± 0.49	60.19 a ± 5.65	6.20 e ± 0.60	60.64 a ± 6.50	7.95 f ± 0.94	71.98 a ± 11.33
Gomisin D	1.08 e ± 0.06	6.48 d ± 0.69	0.55 f ± 0.02	11.02 c ± 1.22	1.19 e ± 0.05	21.45 ab ± 2.05	0.16 f ± 0.02	20.48 b ± 2.20	0.23 f ± 0.03	28.80 a ± 4.53
Gomisin J	0.37 d ± 0.02	3.28 c ± 0.35	0.24 d ± 0.01	7.17 b ± 0.79	0.54 d ± 0.02	13.67 a ± 1.16	traces	10.00 b ± 1.07	0.09 e ± 0.01	15.71 a ± 2.47
Gomisin A	4.60 e ± 0.27	27.66 d ± 2.96	3.00 e ± 0.09	51.10 c ± 5.65	6.03 e ± 0.27	77.84 b ± 7.38	0.74 f ± 0.07	74.62 b ± 8.00	1.32 f ± 0.16	91.53 a ± 14.40
Gomisin G	3.40 b ± 0.20	1.78 d ± 0.19	3.33 b ± 0.10	3.39 b ± 0.38	2.65 c ± 0.12	6.19 a ± 0.57	1.92 d ± 0.19	4.94 a ± 0.53	2.56 c ± 0.30	6.93 a ± 1.09
Licarin B	0.15 a ± 0.01	0.06 c ± 0.01	0.10 b ± 0.003	0.07 c ± 0.01	0.12 b ± 0.01	0.07 c ± 0.01	0.11 b ± 0.01	0.07 c ± 0.01	0.12 b ± 0.01	0.12 b ± 0.02
Epigomisin O	0.60 a ± 0.04	0.07 f ± 0.01	0.43 b ± 0.01	0.13 e ± 0.01	0.35 c ± 0.02	0.27 c ± 0.03	0.24 c ± 0.02	0.21 d ± 0.02	0.35 c ± 0.04	0.29 c ± 0.05
Gomisin O	2.49 a ± 0.15	0.28 f ± 0.03	2.59 a ± 0.08	0.63 e ± 0.1	2.32 a ± 0.10	1.44 b ± 0.13	1.34 b ± 0.13	1.16 b ± 0.12	2.26 a ± 0.27	1.82 b ± 0.27
Mesodihydroguaiaretic acid	traces	traces	0.07 b ± 0.002	0.06 b ± 0.01	traces	0.14 a ± 0.01	traces	0.12 a ± 0.01	traces	0.13 a ± 0.02
Schisantherin A	2.90 a ± 0.17	0.14 e ± 0.01	2.38 b ± 0.07	0.16 e ± 0.02	2.07 b ± 0.09	0.53 ± 0.04	1.29 c ± 0.13	0.32 d ± 0.04	2.05 b ± 0.24	0.46 d ± 0.07
Schisantherin B	1.84 d ± 0.11	3.31 c ± 0.35	1.32 d ± 0.04	6.69 b ± 0.74	1.51 d ± 0.07	11.81 a ± 1.10	0.64 e± 0.06	12.74 a ± 1.37	0.80 e ± 0.09	16.66 a ± 2.62
Licarin A	9.94 b ±0.59	1.65 e ± 0.18	12.30 a ± 0.38	5.81 c ± 0.64	5.19 c± 0.23	3.54 d ± 0.34	2.76 d ± 0.27	2.86 d ± 0.31	9.71 b ± 1.15	1.81 e ± 0.29
Schisanhenol	5.94 a ± 0.35	1.50 d ± 0.16	6.06 a ± 0.19	2.19 c ± 0.24	4.03 b ± 0.18	4.02 b ±0.38	2.35 c ± 0.23	4.09 b ± 0.44	3.59 b ± 0.43	4.74 b ± 0.75
Deoxyschisandrin	11.79 d ± 0.70	34.21 c ± 3.66	8.69 e ± 0.27	53.87 b ± 5.96	10.25 d ± 0.45	85.16 a ± 7.91	3.58 e ± 0.35	91.53 a ± 9.82	4.57 e ± 0.54	93.04 a ± 14.64
Fragransin A	traces	traces	traces	traces	traces	traces	traces	traces	traces	traces
Pregomisin	traces	traces	traces	traces	traces	traces	traces	traces	traces	traces
Gomisin N	2.95 d ± 0.17	6.78 c ± 0.73	2.15 d ± 0.07	13.88 b ± 1.53	2.56 d ± 0.11	23.79 a ± 2.14	0.92 e ± 0.09	22.00 a ± 2.36	1.68 d ± 0.20	28.07 a ± 4.42
6-O-Benzylgomisin O	0.51 b ± 0.03	0.29 c ± 0.03	0.42 b ± 0.01	0.58 b ± 0.06	0.41 b ± 0.02	1.21 a ± 0.11	0.21 c ± 0.02	1.01 a ± 0.11	0.32 c ± 0.04	1.22 a ± 0.19
Schisandrin C	0.19 d ± 0.01	1.44 c ± 0.15	traces	2.98 b ± 0.33	0.19 d ± 0.01	5.24 a ± 0.47	traces	3.54 b ± 0.38	0.06 d ± 0.01	5.17 a ± 0.81
**Total content**	62.80 d ± 3.72	112.10 c ± 11.98	55.50 e ± 1.32	194.10 b ± 1.46	50.60 e ± 2.22	316.65 a ± 29.46	22.60 g ± 2.19	310.40 a ± 33.28	37.80 f ± 4.47	368.50 a ± 57.98

* traces < 0.05 mg/100 g DW.

**Table 3:** Accumulation of lignans (mg/100 g DW) in agitated cultures of *S. rubriflora* lines F and M 48 h after addition of elicitor. Data expressed as the mean value ± SD (*n* = 3). Different superscript letters (a–f) within a row indicate significant differences between means (Duncan’s multiple range test; *p* < 0.05).

Lignan	Control		Elicitor							
			CH		YeE		ETH		MeJA	
	F	M	F	M	F	M	F	M	F	M
Wulignan A1	0.10 a ± 0.01	traces *	0.10 a ± 0.01	0.10 a ± 0.01	traces	traces	0.07 a ± 0.01	traces	0.06 a ± 0.003	traces
Rubrisandrin A	traces	traces	traces	traces	traces	traces	traces	traces	traces	traces
Interiotherin C	traces	traces	0.10 a ± 0.01	0.10 a ± 0.01	traces	traces	traces	traces	traces	traces
Schisandrin	16.67 e ± 1.92	40.74 b ± 4.64	11.20 f ± 1.12	61.85 a ± 6.28	9.83 f ± 1.11	56.37 a ± 4.50	29.61 c ± 3.92	43.15 b ± 3.84	22.21 d ± 1.24	34.25 c ± 3.07
Gomisin D	1.47 e ± 0.17	12.72 b ± 1.45	1.09 e ± 0.11	24.47 a ± 2.48	0.36 f ± 0.04	20.04 a ± 1.60	3.84 d ± 0.51	15.51 b ± 1.38	1.97 e ± 0.11	8.30 c ± 0.74
Gomisin J	0.65 e ± 0.08	7.66 b ± 0.87	0.44 e ± 0.04	11.58 a ± 1.18	0.14 f ± 0.02	10.17 a ± 0.81	1.84 d ± 0.24	7.66 b ± 0.68	0.73 e ± 0.04	5.16 c ± 0.46
Gomisin A	5.66 d ± 0.65	46.22 b ± 5.26	3.84 e ± 0.38	81.59 a ± 8.28	1.38 f ± 0.16	66.50 a ± 5.31	15.90 c ± 2.11	54.04 b ± 4.81	7.42 d ± 0.42	41.11 b ± 3.68
Gomisin G	3.45 b ± 0.40	3.16 bc ± 0.36	3.42 b ± 0.34	5.94 a ± 0.60	3.06 bc ± 0.34	4.26 b ± 0.34	6.53 a ± 0.87	3.84 c ± 0.34	6.04 a ± 0.34	1.92 d ± 0.17
Licarin B	0.12 c ± 0.01	0.11 c ± 0.01	0.14 c ± 0.01	0.14 c ± 0.01	0.15 c ± 0.02	0.14 c ± 0.01	0.41 a ± 0.06	0.24 b ± 0.02	0.21 b ± 0.01	0.08 d ± 0.01
Epigomisin O	0.64 b ± 0.07	0.17 cd ± 0.02	0.59 b ± 0.06	0.24 c ± 0.03	0.43 b ± 0.05	0.22 c ± 0.02	1.54 a ± 0.20	0.27 c ± 0.02	1.00 a ± 0.06	0.12 d ± 0.01
Gomisin O	3.48 b ± 0.40	0.78 e ± 0.09	3.10 b ± 0.31	1.43 d ± 0.15	2.82 c ± 0.32	1.23 d ± 0.10	4.47 a ± 0.59	1.18 d ± 0.11	4.80 a ± 0.27	0.60 e ± 0.05
Mesodihydroguaiaretic acid	traces	0.07 c ± 0.01	0.09 bc ± 0.01	0.09 bc ± 0.01	0.05 cd ± 0.01	0.11 b ± 0.01	0.16 a ± 0.02	0.08 c ± 0.01	0.11 b ± 0.01	0.07 c ± 0.01
Schisantherin A	2.72 c ± 0.31	0.27 e ± 0.03	2.29 c ± 0.23	0.33 de ± 0.03	2.25 c ± 0.25	0.39 d ± 0.03	5.42 a ± 0.72	0.51 d ± 0.05	4.19 b ± 0.23	0.18 e ± 0.02
Schisantherin B	2.06 d ± 0.24	7.78 b ± 0.89	1.54 e ± 0.15	12.57 a ± 1.28	1.21 e ± 0.14	10.65 a ± 0.85	4.73 c ± 0.63	9.45 a ± 0.84	2.82 d ± 0.16	5.35 c ± 0.48
Licarin A	12.94 a ± 1.49	2.53 d ± 0.29	14.85 a ± 1.48	10.50 ab ± 1.07	7.48 b ± 0.84	3.58 c ± 0.29	6.81 b ± 0.90	2.95 d ± 0.26	8.68 b ± 0.49	4.19 c ± 0.38
Schisanhenol	5.65 c ± 0.65	3.52 d ± 0.40	5.74 c ± 0.57	4.14 d ± 0.42	5.60 c ± 0.63	4.02 d ± 0.32	10.11 a ± 1.34	3.24 de ± 0.29	7.97 b ± 0.45	2.28 e ± 0.20
Deoxyschisandrin	15.06 d ± 1.73	67.13 ab ± 7.64	9.05 e ± 0.90	82.87 a ± 8.41	7.40 e ± 0.83	77.42 a ± 6.18	23.55 c ± 3.12	73.33 a ± 6.53	15.86 d ± 0.89	53.01 b ± 4.75
Fragransin A	traces	traces	traces	traces	traces	traces	traces	traces	traces	traces
Pregomisin	traces	traces	traces	traces	traces	traces	traces	traces	traces	traces
Gomisin N	3.00 d ± 0.35	14.14 b ± 1.61	2.26 e ± 0.23	20.49 a ± 2.08	1.86 e ± 0.21	18.56 a ± 1.48	7.57 c ± 1.00	17.89 a ± 1.59	4.55 d ± 0.25	12.04 b ± 1.08
6-O-benzylgomisin O	0.53 c ± 0.06	0.74 b ± 0.08	0.44 c ± 0.04	0.79 b ± 0.08	0.44 c ± 0.05	0.89 b ± 0.07	1.40 a ± 0.19	0.89 b ± 0.08	0.82 b ± 0.05	0.40 c ± 0.04
Schisandrin C	0.23 d ± 0.03	2.74 b ± 0.31	0.16 d ± 0.02	4.16 a ± 0.42	traces	3.58 a ± 0.29	0.52 d ± 0.07	2.86 b ± 0.26	0.24 d ± 0.01	1.88 c ± 0.17
**Total content**	74.50 c ± 8.56	210.60 ab ± 23.98	60.30 c ± 6.03	323.30 a ± 32.81	44.60 d ± 5.01	278.19 a ± 22.22	124.50 b ± 16.50	237.10 a ± 21.11	89.70 c ± 5.01	171.00 b ± 15.31

* traces < 0.05 mg/100 g DW.

**Table 4:** Accumulation of lignans (mg/100 g DW) in agitated cultures of *S. rubriflora* lines F and M 4 days after addition of elicitor. Data expressed as the mean value ± SD (*n* = 3). Different superscript letters (a–f) within a row indicate significant differences between means (Duncan’s multiple range test; *p* < 0.05).

Lignan	Control		Elicitor							
			CH		YeE		ETH		MeJA	
	F	M	F	M	F	M	F	M	F	M
Wulignan A1	0.16 a ± 0.02	0.05 c ± 0.01	0.16 a ± 0.02	0.08 c ± 0.01	0.11 b ± 0.01	traces *	traces	traces	0.12 b ± 0.01	0.06 c ± 0.01
Rubrisandrin A	traces	traces	traces	traces	traces	0.14 ± 0.01	traces	traces	traces	traces
Interiotherin C	traces	traces	0.16 a ± 0.02	0.08 b ± 0.01	traces	traces	traces	traces	traces	traces
Schisandrin	11.17 d ± 1.19	46.47 b ± 4.94	5.82 e ± 0.62	43.38 b ± 3.25	10.43 d ± 0.63	70.80 a ± 5.71	14.90 d ± 0.95	57.02 ab ± 2.45	9.14 de ± 0.80	35.16 c ± 3.30
Gomisin D	1.69 d ± 0.18	17.45 b ± 1.85	0.69 e ± 0.07	15.02 b ± 1.13	1.97 d ± 0.12	25.86 a ± 2.13	0.89 e ± 0.06	22.50 a ± 0.97	0.45 e ± 0.04	10.59 c ± 0.99
Gomisin J	0.56 d ± 0.06	6.72 bc ± 0.71	0.29 f ± 0.03	8.84 b ± 0.66	0.78 d ± 0.05	14.11 a ± 1.19	0.57 d ± 0.04	14.59 a ± 0.63	0.26 f ± 0.02	5.00 c ± 0.470
Gomisin A	6.15 c ± 0.65	55.88 b ± 5.94	2.92 d ± 0.31	56.64 b ± 4.25	8.05 c ± 0.49	85.79 a ± 6.96	5.78 c ± 0.37	82.32 a ± 3.54	2.59 d ± 0.23	44.74 b ± 4.20
Gomisin G	2.59 c ± 0.28	4.31 b ± 0.46	1.95 d ± 0.21	3.75 b ± 0.28	2.59 c ± 0.16	6.53 a ± 0.55	4.93 b ± 0.31	6.76 a ± 0.29	2.79 c ± 0.25	2.71 c ± 0.25
Licarin B	0.16 a ± 0.02	0.05 c ± 0.01	0.05 c ± 0.01	traces	0.07 b ± 0.004	0.15 a ± 0.01	0.17 a ± 0.01	0.10 b ± 0.004	0.09 b ± 0.01	0.09 b ± 0.01
Epigomisin O	0.38 b ± 0.04	0.19 c ± 0.02	0.23 bc ± 0.03	0.14 c ± 0.01	0.28 b ± 0.02	0.27 b ± 0.02	0.93 a ± 0.06	0.26 b ± 0.01	0.26 b ± 0.02	0.12 c ± 0.01
Gomisin O	1.84 b ± 0.20	0.84 c ± 0.09	1.42 b ± 0.15	0.76 c ± 0.06	1.58 b ± 0.10	1.47 b ± 0.12	3.73 a ± 0.24	1.51 b ± 0.07	1.34 bc ± 0.12	0.55 c ± 0.05
Meso-dihydroguaiaretic acid	traces	0.07 b ± 0.01	traces	0.09 b ± 0.01	0.06 b ± 0.003	0.15 a ± 0.01	0.06 b ± 0.004	0.13 a ± 0.01	0.06 b ± 0.01	0.07 b ± 0.01
Schisantherin A	1.80 b ± 0.19	0.25 c ± 0.03	1.43 b ± 0.15	0.16 e ± 0.01	1.78 b ± 0.11	0.47 c ± 0.04	3.10 a ± 0.20	0.58 c ± 0.03	1.66 b ± 0.15	0.15 e ± 0.01
Schisantherin B	1.50 c ± 0.16	7.46 b ± 0.79	0.88 d ± 0.09	7.50 b ± 0.56	1.61 c ± 0.10	13.34 a ± 1.10	2.16 c ± 0.14	15.17 a ± 0.65	0.89 d ± 0.08	6.43 b ± 0.60
Licarin A	14.58 c ± 1.55	4.30 e ± 0.46	20.41 b ± 2.15	10.12 d ± 0.76	16.32 c ± 0.99	3.46 e ± 0.26	3.10 e ± 0.20	6.14 de ± 0.26	30.83 a ± 2.07	3.88 e ± 0.36
Schisanhenol	4.17 b ± 0.44	2.87 d ± 0.31	3.32 c ± 0.35	2.82 d ± 0.21	3.94 c ± 0.24	4.67 b ± 0.38	6.32 a ± 0.40	4.43 b ± 0.19	2.94 d ± 0.26	2.11 d ± 0.20
Deoxyschisandrin	11.17 c ± 1.19	57.36 b ± 6.09	6.61 d ± 0.70	58.91 b ± 4.42	11.45 c ± 0.70	94.86 a ± 7.56	11.58 c ± 0.74	91.58 a ± 3.93	6.49 d ± 0.57	55.33 b ± 5.19
Fragransin A	traces	traces	traces	traces	traces	traces	traces	traces	traces	traces
Pregomisin	traces	traces	traces	traces	traces	traces	traces	traces	traces	traces
Gomisin N	2.08 d ± 0.22	13.07 b ± 1.39	1.53 de ± 0.16	13.65 b ± 1.02	2.46 d ± 0.15	24.89 a ± 2.05	3.48 c ± 0.22	25.71 a ±0.01	1.63 de ± 0.14	12.52 b ± 1.17
6-O-Benzylgomisin O	0.37 c ± 0.04	0.63 b ± 0.07	0.28 c ± 0.03	0.56 b ± 0.04	0.35 c ± 0.02	1.12 a ± 0.09	0.82 a ± 0.05	1.21 a ± 0.05	0.34 c ± 0.03	0.52 b ± 0.05
Schisandrin C	0.23 c ± 0.03	2.50 b ± 0.27	0.12 ce ± 0.01	2.77 b ± 0.21	0.30 c ± 0.02	5.05 a ± 0.42	0.19 c ± 0.01	4.81 a ± 0.21	0.07 e ± 0.01	1.94 b ± 0.18
**Total content**	60.70 c ± 6.44	220.50 b ± 23.43	48.20 d ± 5.09	225.20 b ± 6.88	64.10 c ± 3.90	353.17 a ± 28.62	62.80 c ± 4.00	334.90 a ± 14.39	61.90 c ± 4.80	182.00 b ± 17.07

* traces < 0.05 mg/100 g DW.

**Table 5:** Accumulation of lignans (mg/100 g DW) in agitated cultures of *S. rubriflora* lines F and M 6 days after addition of elicitor. Data expressed as the mean value ± SD (*n* = 3). Different superscript letters (a–f) within a row indicate significant differences between means (Duncan’s multiple range test; *p* < 0.05).

Lignan	Control		Elicitor							
			CH		YeE		ETH		MeJA	
	F	M	F	M	F	M	F	M	F	M
Wulignan A1	0.15 b ± 0.02	0.06 c ± 0.01	0.36 a ± 0.04	0.11 b ± 0.01	0.26 a ± 0.02	traces *	traces	traces	0.11 b ± 0.01	traces
Rubrisandrin A	traces	traces	traces	traces	traces	0.19 ± 0.01	traces	traces	traces	traces
Interiotherin C	traces	traces	0.36 a ± 0.04	0.11 b ± 0.01	traces	traces	traces	traces	traces	traces
Schisandrin	16.52 c ± 2.11	36.64 b ± 4.89	16.20 c ± 1.69	46.50 a ± 3.25	14.74 c ± 0.88	57.94 a ± 4.13	13.74 c ± 0.84	25.62 bc ± 1.98	14.84 c ± 1.42	28.92 b ± 1.23
Gomisin D	1.45 e ± 0.19	11.97 b ± 1.60	1.48 e ± 0.15	15.18 b ± 1.06	2.84 d ± 0.17	20.12 a ± 1.44	1.49 e ± 0.09	7.04 c ± 0.54	0.59 f ± 0.06	11.42 b ± 0.49
Gomisin J	0.37 e ± 0.05	3.19 bc ± 0.43	0.60 e ± 0.06	8.18 a ± 0.57	1.47 d ± 0.09	12.37 a ± 0.88	0.64 e ± 0.04	2.69 c ± 0.21	0.30 e ± 0.03	4.76 b ± 0.20
Gomisin A	5.06 e ± 0.65	42.04 b ± 5.61	5.62 e ± 0.59	53.51 a ± 3.74	11.32 d ± 0.68	68.37 a ± 4.87	7.04 de ± 0.42	30.24 c ± 2.33	3.24 f ± 0.31	38.78 b ± 1.65
Gomisin G	3.99 a ± 0.51	2.83 b ± 0.38	3.13 b ± 0.33	3.81 a ± 0.27	3.06 b ± 0.18	4.74 a ± 0.34	3.43 b ± 0.21	1.50 c ± 0.12	3.97 a ± 0.38	3.03 b ± 0.13
Licarin B	0.26 a ± 0.03	0.18 b ± 0.02	0.11 c ± 0.01	0.15 b ± 0.01	traces	0.06 c ± 0.004	0.22 a ± 0.01	0.11 c ± 0.01	0.28 a ± 0.03	traces
Epigomisin O	0.61 a ± 0.08	0.15 c ± 0.02	0.57 a ± 0.06	0.17 c ± 0.01	0.36 b ± 0.02	0.20 c ± 0.01	0.55 a ± 0.03	0.08 d ± 0.01	0.49 a ± 0.05	0.10 d ± 0.004
Gomisin O	2.88 a ± 0.37	0.55 e ± 0.07	2.78 a ± 0.30	0.88 ce ± 0.06	2.301 b ± 0.14	1.10 c ± 0.08	3.04 a ± 0.19	0.38 e ± 0.03	2.64 a ± 0.25	0.62 e ± 0.03
Meso-dihydroguaiaretic acid	traces	traces	0.08 ± 0.01	0.10 ± 0.01	0.07 ab ± 0.004	0.11 a ± 0.01	0.06 b ± 0.004	traces	0.08 a ± 0.01	0.06 b ± 0.003
Schisantherin A	2.89 a ± 0.37	0.20 c ± 0.03	2.33 b ± 0.24	0.35 c ± 0.03	2.21 b ± 0.13	0.31 c ± 0.02	2.42 a ± 0.15	0.10 c ± 0.01	2.77 a ± 0.27	0.14 c ± 0.01
Schisantherin B	1.99 c ± 0.25	5.35 b ± 0.71	1.85 c ± 0.19	10.04 a ± 0.70	2.12 c ± 0.13	10.69 a ± 0.76	2.09 c ± 0.13	4.23 b ± 0.33	1.57 d ± 0.15	5.60 b ± 0.24
Licarin A	22.54 b ± 2.87	4.83 d ± 0.64	34.71 a ± 3.62	11.52 c ± 0.81	35.38 a ± 2.11	3.27 d ± 0.23	5.48 d ± 0.33	3.56 d ± 0.28	9.64 c ± 0.93	1.80 e ± 0.08
Schisanhenol	6.84 a ± 0.87	2.35 c ± 0.31	6.94 a ± 0.72	3.43 b ± 0.24	5.53 a ± 0.33	4.08 b ± 0.29	5.74 a ± 0.35	1.74 c ± 0.13	5.95 a ± 0.57	1.80 c ± 0.08
Deoxyschisandrin	15.20 c ± 1.94	49.24 b ± 6.57	14.50 c ± 1.51	77.72 a ± 5.44	17.40 c ± 1.04	80.44 a ± 5.73	14.19 c ± 0.86	39.93 b ± 3.08	13.13 c ± 1.26	43.25 b ± 1.84
Fragransin A	traces	traces	traces	traces	traces	traces	traces	traces	traces	traces
Pregomisin	traces	traces	traces	traces	traces	traces	traces	traces	traces	traces
Gomisin N	2.53 d ± 0.32	9.49 b ± 1.27	3.08 d ± 0.32	18.82 a ± 1.32	3.88 d ± 0.23	20.49 a ± 1.46	3.21 d ± 0.20	7.60 c ± 0.59	2.90 d ± 0.28	10.24 b ± 0.44
6-O-Benzylgomisin O	0.56 b ± 0.07	0.40 b ± 0.05	0.49 b ± 0.05	0.71 ab ± 0.05	0.48 b ± 0.03	1.12 a ± 0.08	0.51 b ± 0.03	0.29 c ± 0.02	0.57 b ± 0.06	0.40 bc ± 0.02
Schisandrin C	0.20 e ± 0.01	1.99 b ± 0.27	0.19 e ± 0.02	3.17 a ± 0.22	0.52 de ± 0.03	3.93 a ± 0.28	0.24 e ± 0.02	1.10 c ± 0.09	0.10 f ± 0.01	1.67 b ± 0.07
**Total content**	84.10 cd ± 10.72	171.60 b ± 22.90	95.00 c ± 9.95	254.40 a ± 17.81	103.90 c ± 6.20	289.57 a ± 20.63	64.10 d ± 3.90	126.30 b ± 9.74	63.20 d ± 6.07	152.70 b ± 6.50

* traces < 0.05 mg/100 g DW.

**Table 6:** Accumulation of lignans (mg/100 g DW) in agitated cultures of *S. rubriflora* lines F and M 8 days after addition of elicitor. Data expressed as the mean value ± SD (*n* = 3). Different superscript letters (a–f) within a row indicate significant differences between means (Duncan’s multiple range test; *p* < 0.05).

Lignan	Control		Elicitor							
			CH		YeE		ETH		MeJA	
	F	M	F	M	F	M	F	M	F	M
Wulignan A1	0.09 b ± 0.01	0.05 b ± 0.003	0.31 a ± 0.02	0.08 b ± 0.01	0.31 a ± 0.04	traces *	traces	traces	traces	0.05 ± 0.01
Rubrisandrin A	traces	traces	traces	traces	traces	0.15 ± 0.01	traces	traces	traces	traces
Interiotherin C	traces	traces	traces	traces	traces	traces	traces	traces	traces	traces
Schisandrin	11.69 d ± 1.02	31.38 b ± 1.95	8.27 d ± 0.67	37.83 b ± 2.39	17.49 c ± 2.02	47.87 a ± 3.68	14.52 cd ± 1.05	29.40 b ± 2.14	10.22 d ± 0.92	56.95 a ± 5.09
Gomisin D	1.38 d ± 0.12	10.00 b ± 0.62	0.57 e ± 0.05	12.26 b ± 0.78	1.66 d ± 0.2	17.35 a ± 1.33	1.11 d ± 0.08	6.91 c ± 0.50	0.77 e ± 0.07	18.53 a ± 1.66
Gomisin J	0.36 d ± 0.03	3.52 c ± 0.22	0.22 e ± 0.02	5.79 b ± 0.37	0.63 d ± 0.08	8.90 a ± 0.69	0.44 d ± 0.03	2.97 c ± 0.22	0.35 d ± 0.07	9.23 a ± 0.83
Gomisin A	4.77 d ± 0.42	37.39 c ± 2.33	1.77 f ± 0.15	45.03 b ± 2.84	5.41 d ± 0.65	58.65 a ± 4.51	5.86 d ± 0.42	37.00 c ± 2.69	3.72 e ± 0.33	69.87 a ± 6.24
Gomisin G	3.35 b ± 0.29	2.46 c ± 0.15	2.71 c ± 0.22	2.66 c ± 0.17	4.08 a ± 0.49	4.25 a ± 0.33	4.31 a ± 0.31	2.07 d ± 0.15	3.02 b ± 0.27	4.52 a ± 0.40
Licarin B	0.14 c ± 0.01	0.14 c ± 0.01	0.09 d ± 0.01	0.13 c ± 0.01	0.16 b ± 0.02	0.07 d ± 0.01	0.15 bc ± 0.01	0.14 c ± 0.01	0.19 a ± 0.02	0.10 d ± 0.01
Epigomisin O	0.52 b ± 0.05	0.18 d ± 0.01	0.33 c ± 0.03	0.12 e ± 0.01	0.69 a ± 0.08	0.19 d ± 0.02	0.54 b ± 0.04	0.11 e ± 0.01	0.39 c ± 0.04	0.19 d ± 0.02
Gomisin O	2.68 b ± 0.23	0.76 d ± 0.05	1.84 c ± 0.15	0.61 de ± 0.04	3.36 a ± 0.41	0.83 d ± 0.06	2.70 b ± 0.19	0.55 e ± 0.04	2.37 b ± 0.21	0.93 d ± 0.08
Mesodihydroguaiaretic acid	traces	traces	0.07 c ± 0.01	0.07 c ± 0.01	0.09 b ± 0.01	0.07 c ± 0.01	0.06 c ± 0.004	traces	traces	0.23 a ± 0.02
Schisantherin A	2.64 a ± 0.23	0.29 e ± 0.02	1.38 c ± 0.11	0.19 f ± 0.01	2.49 b ± 0.27	0.22 e ± 0.02	3.01 a ± 0.22	0.18 f ± 0.01	2.11 b ± 0.19	0.42 d ± 0.04
Schisantherin B	1.84 d ± 0.16	5.09 c ± 0.32	1.00 e ± 0.08	7.45 bc ± 0.47	2.18 d ± 0.26	9.02 b ± 0.69	1.89 d ± 0.14	4.98 c ± 0.36	1.28 e ± 0.12	10.21 a ± 0.91
Licarin A	11.61 b ± 1.01	6.06 c ± 0.38	31.81 a ± 2.60	8.97 bc ± 0.56	37.54 a ± 4.51	2.68 e ± 0.21	5.39 d ± 0.39	2.84 e ± 0.21	5.04 d ± 0.45	4.04 d ± 0.36
Schisanhenol	5.36 b ± 0.47	2.15 d ± 0.15	4.79 b ± 0.39	2.48 d ± 0.16	7.52 a ± 0.88	3.31 cd ± 0.25	4.44 c ± 0.32	2.03 d ± 0.15	5.12 b ± 0.46	3.30 cd ± 0.30
Deoxyschisandrin	12.53d ± 1.09	44.70 c ± 2.80	7.39 e ± 0.60	59.76 bc ± 3.76	16.14 d ± 1.90	69.68 a ± 5.34	10.53 d ±0.76	49.35 c ± 3.60	10.08 de ±0.91	80.06 a ± 7.15
Fragransin A	traces	traces	traces	traces	traces	traces	traces	traces	traces	traces
Pregomisin	traces	traces	traces	traces	traces	traces	traces	traces	traces	traces
Gomisin N	2.82 ef ± 0.25	9.14 c ± 0.57	1.55 ± 0.13	13.85 b ± 0.88	3.67 e ± 0.43	17.52 a ± 1.34	3.11 e ± 0.22	10.33 bc ± 0.75	2.05 f ± 0.18	19.55 a ± 1.75
6-O-benzylgomisin O	0.54 b ± 0.05	0.43 e ± 0.03	0.33 f ± 0.03	0.49 be ± 0.03	0.64 b ± 0.08	0.85 a ± 0.07	0.54 b ± 0.04	0.42 e ± 0.03	0.38 e ± 0.03	0.83 a ± 0.07
Schisandrin C	0.24 e ± 0.02	1.72 bc ± 0.11	0.08 f ± 0.01	2.43 b ± 0.15	0.24 e ± 0.03	3.44 a ± 0.27	0.17 e ± 0.01	1.47 c ± 0.11	0.15 e ± 0.01	3.08 a ± 0.28
**Total content**	62.70 e ± 5.45	155.77 c ± 9.71	64.55 e ± 5.24	200.21 b ± 12.62	104.29 d ± 12.35	245.13 a ± 18.83	58.80 e ± 4.23	150.80 c ± 10.97	47.33 f ± 4.26	47.30 f ± 25.21

* traces < 0.05 mg/100 g DW.

**Table 7:** Comparison of dibenzocyclooctadiene lignans content and elicitation conditions in microshoot, agitated in vitro cultures of *S. rubriflora* and *S. chinensis* species [22].

Dibenzocyclooctadiene Lignans	*S. rubriflora*			*S. chinensis*		
	Maximal Content (mg/100 g DW)	Elicitation Conditions	The Fold Increase in Content Compared to the Control	Maximal Content (mg/100 g DW)	Elicitation Conditions	The Fold Increase in Content Compared to the Control
Schisandrin	71.98 ± 11.33	MeJA, 50 µM, 24 h	3.13×	183.60 ± 9.10	CdCl_2_, 1000 µM, 10 days	2.28×
Gomisin A	91.53 ± 14.40	MeJA, 50 µM, 24 h	3.31×	115.90 ± 17.00	CdCl_2_, 1000 µM, 10 days	2.20×
Gomisin G	6.93 ± 1.09	MeJA, 50 µM, 24 h	3.91×	11.80 ± 1.30	YeE, 3000 mg/L, days 0	3.03×
Schisantherin A	5.42 ± 0.72	ETH, 25 µM, 48 h	1.99×	5.60 ± 0.10	YeE, 3000 mg/L, days 0	3.11×
Schisantherin B	16.66 ± 2.62	MeJA, 50 µM, 24 h	5.03×	28.20 ± 0.5	YeE, 1000 mg/L, 20 days	2.61×
Schisanhenol	10.11 ± 1.34	ETH, 25 µM, 48 h	1.79×	11.10 ± 0.70	CdCl_2_, 1000 µM, 10 days	4.44×
Deoxyschisandrin	94.86 ± 7.56	YeE, 3000 mg/L, 4 days	1.65×	67.4 ± 5.10	YeE, 5000 mg/L, days 0	1.95×
**Total content**	368.50 ± 57.98	MeJA, 50 µM, 24h	3.29×	730.60 ± 53.80	CdCl_2_, 1000 µM, 10 days	2.0×

**Table 8:** Content (mg/100 g DW ± SD) of lignans in extracts from *S. rubriflora* microshoot cultures grown in Plantform bioreactors; control and elicited. Data expressed as the mean value ± SD (*n* = 3). Different superscript letters (a–c) within a row indicate significant differences between means (Duncan’s multiple range test; *p* < 0.05).

Lignan	Control	Elicitor			
		CH	YeE	ETH	MeJA
Wulignan A1	traces *	traces	traces	traces	traces
Rubrisandrin A	traces	traces	0.08 ± 0.01	traces	traces
Interiotherin C	traces	traces	traces	traces	traces
Schisandrin	30.10 a ± 2.90	28.7 a ± 4.50	30.00 a ± 4.00	25.00 b ± 1.4	37.6 a ± 4.00
Gomisin D	10.7 a ± 1.00	9.60 ab ± 1.50	9.8 a ± 1.30	9.00 b ± 0.50	11.30 a ± 1.20
Gomisin J	3.45 b ± 0.33	2.86 bc ± 0.45	3.07 b ± 0.41	2.77 c ± 0.15	4.40 a ± 0.47
Gomisin A	39.98 a ± 3.80	34.00 ab ± 5.30	35.06 ab ± 4.60	31.42 b ± 1.80	41.01 a ± 4.40
Gomisin G	3.16 a ± 0.30	2.48 b ± 0.40	2.62 ab ± 0.30	2.36 b ± 0.10	3.33 a ± 0.40
Licarin B	traces	traces	traces	traces	traces
Epigomisin O	0.10 a ± 0.01	0.10 a ± 0.01	0.09 ab ± 0.01	0.08 b ± 0.01	0.11 a ± 0.01
Gomisin O	0.40 a ± 0.04	0.34 b ± 0.05	0.35 b ± 0.05	0.30 c ± 0.02	0.42 a ± 0.04
Mesodihydroguaiaretic acid	traces	traces	traces	traces	traces
Schisantherin A	0.08 b ± 0.01	0.08 b ± 0.01	0.08 b ± 0.01	30.00 a ± 0.06	0.09 b ± 0.01
Schisantherin B	4.50 a ± 0.40	4.40 ab ± 0.70	4.30 ab ± 0.60	3.60 b ± 0.20	4.60 a ± 0.50
Licarin A	0.58 b ± 0.06	0.85 a ± 0.13	0.51 b ± 0.07	0.38 c ± 0.02	0.47 b ± 0.05
Schisanhenol	1.30 a ± 0.10	1.30 a ± 0.20	1.30 a ± 0.20	1.10 a ± 0.10	1.30 a ± 0.10
Deoxyschisandrin	33.00 a ± 3.00	35.00 a ± 6.00	37.00 a ± 5.00	29.00 b ± 2.00	35.00 a ± 4.00
Fragransin A	traces	traces	traces	traces	traces
Pregomisin	traces	traces	traces	traces	traces
Gomisin N	7.30 a ± 0.70	7.50 a ± 1.20	7.90 a ± 1.10	5.9 b ± 0.30	7.80 a ± 0.80
6-O-Benzylgomisin O	0.70 ab ± 0.10	0.80 a ± 0.10	0.80 a ± 0.10	0.6 b ± 0.01	0.80 a ± 0.10
Schisandrin C	4.60 a ± 0.40	4.20 ab ± 0.70	4.40 a ± 0.60	3.60 b ± 0.20	5.10 a ± 0.50
**Total content**	136.00 ab ± 3.06	132.40 b ± 21.25	137.80 ab ± 18.36	114.80 c ± 6.87	153.20 a ± 16.58

* traces < 0.05 mg/100 g DW.

**Table 9:** Comparison of maximum lignan contents (mg/100 g DW) obtained from elicitation experiments on microshoot cultures of lines F and M with their contents in the fruits, leaves and shoots of the parent plant *S. rubriflora* [11].

Lignans	In Vitro Cultures After Elicitor Treatment			Parent Plant		
	Maximal Content	Elicitation Conditions	The Fold Increase in Content Compared to the Control	Fruits	Leaves	Stems
Wulignan A1	0.36 ± 0.04	CH 50 µM, 6 days, line F	2.37×	19.39	0.04 (F)	0.03 (F)
Rubrisandrin A	0.19 ± 0.01	YeE 3000 mg/L, 6 days, line F	3.82×	0.07	0.06 (F)	0.1 (F)
Interiotherin C	0.36 ± 0.04	CH 50 µM, 6 days, line F	7.16×	traces *	traces	traces
Schisandrin	71.98 ± 11.33	MeJA 50 µM, 24h, line M	3.13×	6.57	5.69 (M)	2.25 (M)
Gomisin D	28.80 ± 4.53	MeJA 50 µM, 24h, line M	4.44×	3.52	116.51 (M)	20.26 (M)
Gomisin J	15.71 ± 2.47	MeJA 50 µM, 24h, line M	4.78×	5.4	0.76 (M)	0.36 (M)
Gomisin A	91.53 ± 14.40	MeJA 50 µM, 24h, line M	3.31×	0.75	4.20 (M)	1.65 (M)
Gomisin G	6.93 ± 1.09	MeJA 50 µM, 24h, line M	3.91×	66.39	8.23 (M)	3.67 (M)
Licarin B	0.41 ± 0.06	ETH 25 µM, 48h, line F	3.33×	1.98	0.41 (F)	0.19 (F)
Epigomisin O	1.54 ± 0.20	ETH 25 µM, 48h, line F	2.40×	7.46	10.62 (F)	4.91 (F)
Gomisin O	4.47 ± 0.59	ETH 25 µM, 48h, line F	1.38×	103.64	22.9 (F)	12.07 (F)
Mesodihydroguaiaretic acid	0.23 ± 0.02	MeJA 50 µM, 8 days, line M	4.62×	1.03	0.32 (M)	0.16 (M)
Schisantherin A	30.00 ± 0.06	ETH 25 µM, 4 days, line M bioreactor Plantform	4.35×	27.19	226.8 (M)	84.35 (M)
Schisantherin B	16.66 ± 2.62	MeJA 50 µM, 24 h, line M	5.03×	118.07	104.28 (M)	169.04 (M)
Licarin A	37.54 ± 4.51	YeE 3000 mg/L, 8 days, line F	3.23×	0.41	0.73 (F)	0.33 (F)
Schisanhenol	10.11 ± 1.34	ETH 25 µM, 48 h, line F	1.79×	268.02	2.05 (F)	1.13 (F)
Deoxyschisandrin	94.86 ± 7.56	YeE 3000 mg/L, 4 days, line M	1.65×	104.32	0.38 (M)	0.5 (M)
Gomisin N	28.07 ± 4.42	MeJA 50 µM, 24 h, line M	4.14×	19.2	2.21 (M)	1.08 (M)
6-O-Benzylgomisin O	1.40 ± 0.19	ETH 25 µM, 48 h, line F	2.64×	35.28	134.51 (F)	72.38 (F)
Schisandrin C	5.24 ± 0.47	YeE 3000 mg/L, 24 h, line M	3.64×	4.96	0.19 (M)	0.09 (M)
**Total content**	368.50 ± 57.98	MeJA 50 µM, 24h, line M	3.29×	793.67 (M)	928.72 (M)	283.54 (M)

* traces < 0.05 mg/100 g DW.

## Data Availability

Not applicable.

## Supplementary Materials

The following supporting information can be downloaded at: https://www.mdpi.com/article/10.3390/molecules27196681/s1, Table S1: The lignan production (mg/100 g DW ± SD) in experimental F and M lines of *S. rubriflora* microshoot cultures-control; traces = <0.05 mg/100 g DW. Table S2: The lignan production (mg/100 g DW ± SD) in experimental F and M lines of *S. rubriflora* microshoot cultures after elicitation with chitosan; traces = <0.05 mg/100 g DW. Table S3: The lignan production (mg/100 g DW ± SD) in experimental F and M lines of *S. rubriflora* microshoot cultures after elicitation with yeast extract; traces = <0.05 mg/100 g DW. Table S4: The lignan production (mg/100 g DW ± SD) in experimental F and M lines of *S. rubriflora* microshoot cultures after elicitation with ethephon; traces = <0.05 mg/100 g DW. Table S5: The lignan production (mg/100 g DW ± SD) in experimental F and M lines of *S. rubriflora* microshoot cultures after elicitation with methyl jasmonate; traces = <0.05 mg/100 g DW.
