# Supplementary material for: Stimulation of Lignan Production in Schisandra rubriflora In Vitro Cultures by Elicitation
Source: Molecules. 2022 Oct 7;27(19):6681. doi: 10.3390/molecules27196681 (PMC9572178; doi:10.3390/molecules27196681)
Supplement: Supplementary file 1 [file molecules-27-06681-s001.zip › molecules-1903648-supplementary.pdf]

SUPPLEMENTARY DATA

Stimulation of Lignan Production in *Schisandra rubriflora* In Vitro Cultures by Elicitation

Agnieszka Szopa [1,*], Michał Dziurka [2], Paweł Kubica [1], Karolina Jafernik [1], Oliwia Siomak [1] and Halina Ekiert [1]

[1] Chair and Department of Pharmaceutical Botany, Jagiellonian University, Medical College, ul. Medyczna 9, 30-688 Kraków, Poland
[2] Polish Academy of Sciences The Franciszek Górski Institute of Plant Physiology, ul. Niezapominajek 21, 30-239 Kraków, Poland
* Correspondence: a.szopa@uj.edu.pl; Tel.: +48-12-620-54-30; Fax: +48-620-54-40

Table S1. The lignan production (mg/100 g DW±SD) in experimental F and M lines of *S. rubriflora* microshoot cultures - control; traces = <0.05 mg/100 g DW.

Lignans	Time of biomass harvesting (without elicitation)									
	24h		48h		4 days		6 days		8 days	
	F	M	F	M	F	M	F	M	F	M
**Wulignan A1**	0.10 ± 0.01	0.02 ± 0.002	0.10 ± 0.01	traces*	0.16 ± 0.02	0.05 ± 0.01	0.15 ± 0.02	0.06 ± 0.01	0.09 ± 0.01	0.05 ± 0.003
**Rubisandrin A**	traces	0.06 ± 0.01	traces	traces	traces	traces	traces	traces	traces	traces
**Intheriotherin C**	traces	traces	traces	traces	traces	traces	traces	traces	traces	traces
**Schisandrin**	13.90 ± 0.82	23.00 ± 2.46	16.67 ± 1.92	40.74 ± 4.64	11.17 ± 1.19	46.47 ± 4.94	16.52 ± 2.11	36.64 ± 4.89	11.69 ± 1.02	31.38 ± 1.95
**Gomisin D**	1.08 ± 0.06	6.48 ± 0.69	1.47 ± 0.17	12.72 ± 1.45	1.69 ± 0.18	17.45 ± 1.85	1.45 ± 0.19	11.97 ± 1.60	1.38 ± 0.12	10.00 ± 0.62
**Gomisin J**	0.37 ± 0.02	3.28 ± 0.35	0.65 ± 0.08	7.66 ± 0.87	0.56 ± 0.06	6.72 ± 0.71	0.37 ± 0.05	3.19 ± 0.43	0.36 ± 0.03	3.52 ± 0.22
**Gomisin A**	4.60 ± 0.27	27.66 ± 2.96	5.66 ± 0.65	46.22 ± 5.26	6.15 ± 0.65	55.88 ± 5.94	5.06 ± 0.65	42.04 ± 5.61	4.77 ± 0.42	37.39 ± 2.33
**Gomisin G**	3.40 ± 0.20	1.78 ± 0.19	3.45 ± 0.40	3.16 ± 0.36	2.59 ± 0.28	4.31 ± 0.46	3.99 ± 0.51	2.83 ± 0.38	3.35 ± 0.29	2.46 ± 0.15
**Licarin B**	0.15 ± 0.01	0.06 ± 0.01	0.12 ± 0.01	0.11 ± 0.01	0.16 ± 0.02	0.05 ± 0.01	0.26 ± 0.03	0.18 ± 0.02	0.14 ± 0.01	0.14 ± 0.01
**Epigomisin O**	0.60 ± 0.04	0.07 ± 0.01	0.64 ± 0.07	0.17 ± 0.02	0.38 ± 0.04	0.19 ± 0.02	0.61 ± 0.08	0.15 ± 0.02	0.52 ± 0.05	0.18 ± 0.01
**Gomisin O**	2.49 ± 0.15	0.28 ± 0.03	3.48 ± 0.40	0.78 ± 0.09	1.84 ± 0.20	0.84 ± 0.09	2.88 ± 0.37	0.55 ± 0.07	2.68 ± 0.23	0.76 ± 0.05
**Meso-dihydroguaiaretic acid**	traces	traces	traces	0.07 ± 0.01	traces	0.07 ± 0.01	traces	traces	traces	traces
**Schisantherin A**	2.90 ± 0.17	0.14 ± 0.01	2.72 ± 0.31	0.27 ± 0.03	1.80 ± 0.19	0.25 ± 0.03	2.89 ± 0.37	0.20 ± 0.03	2.64 ± 0.23	0.29 ± 0.02
**Schisantherin B**	1.84 ± 0.11	3.31 ± 0.35	2.06 ± 0.24	7.78 ± 0.89	1.50 ± 0.16	7.46 ± 0.79	1.99 ± 0.25	5.35 ± 0.71	1.84 ± 0.16	5.09 ± 0.32
**Licarin A**	9.94 ±0.59	1.65 ± 0.18	12.94 ± 1.49	2.53 ± 0.29	14.58 ± 1.55	4.30 ± 0.46	22.54 ± 2.87	4.83 ± 0.64	11.61 ± 1.01	6.06 ± 0.38
**Schisanhenol**	5.94 ± 0.35	1.50 ± 0.16	5.65 ± 0.65	3.52 ± 0.40	4.17 ± 0.44	2.87 ± 0.31	6.84 ± 0.87	2.35 ± 0.31	5.36 ± 0.47	2.15 ± 0.15
**Deoxyschisandrin**	11.79 ± 0.70	34.21 ± 3.66	15.06 ± 1.73	67.13 ± 7.64	11.17 ± 1.19	57.36 ± 6.09	15.20 ± 1.94	49.24 ± 6.57	12.53 ± 1.09	44.70 ± 2.80
**Fragransin A**	traces	traces	traces	traces	traces	traces	traces	traces	traces	traces
**Pregomisin**	traces	traces	traces	traces	traces	traces	traces	traces	traces	traces
**Gomisin N**	2.95 ± 0.17	6.78 ± 0.73	3.00 ± 0.35	14.14 ±1.61	2.08 ± 0.22	13.07 ± 1.39	2.53 ± 0.32	9.49 ± 1.27	2.82 ± 0.25	9.14 ± 0.57
**6-O-benzylgomisin O**	0.51 ± 0.03	0.29 ± 0.03	0.53 ± 0.06	0.74 ± 0.08	0.37 ± 0.04	0.63 ± 0.07	0.56 ± 0.07	0.40 ± 0.05	0.54 ± 0.05	0.43 ± 0.03
**Schisandrin C**	0.19 ± 0.01	1.44 ± 0.15	0.23 ± 0.03	2.74 ± 0.31	0.23 ± 0.03	2.50 ± 0.27	0.20 ± 0.01	1.99 ± 0.27	0.24 ± 0.02	1.72 ± 0.11
**Total content**	**62.80 ± 3.72**	**112.10 ± 11.98**	**74.50 ± 8.56**	**210.60 ± 23.98**	**60.70 ± 6.44**	**220.50 ± 23.43**	**84.10 ± 10.72**	**171.60 ± 22.90**	**62.70 ± 5.45**	**155.77 ± 9.71**

Table S2. The lignan production (mg/100 g DW±SD) in experimental F and M lines of *S. rubriflora* microshoot cultures after elicitation with chitosan; traces = <0.05 mg/100 g DW.

**Lignans**	**Time of biomass harvesting after elicitation**									
	**24h**		**48h**		**4 days**		**6 days**		**8 days**	
	**F**	**M**	**F**	**M**	**F**	**M**	**F**	**M**	**F**	**M**
**Wulignan A1**	0.11 ± 0.003	0.05 ± 0.01	0.10 ± 0.01	0.10 ± 0.01	0.16 ± 0.02	0.08 ± 0.01	0.36 ± 0.04	0.11 ± 0.01	0.31 ± 0.02	0.08 ± 0.01
**Rubisandrin A**	traces	traces	traces	traces	traces	traces	traces	traces	traces	traces
**Intheriotherin C**	0.11 ± 0.003	0.05 ± 0.01	0.10 ± 0.01	0.10 ± 0.01	0.16 ± 0.02	0.08 ± 0.01	0.36 ± 0.04	0.11 ± 0.01	traces	traces
**Schisandrin**	11.64 ± 0.36	34.26 ± 3.79	11.20 ± 1.12	61.85 ± 6.28	5.82 ± 0.62	43.38 ± 3.25	16.20 ± 1.69	46.50 ± 3.25	8.27 ± 0.67	37.83 ± 2.39
**Gomisin D**	0.55 ± 0.02	11.02 ± 1.22	1.09 ± 0.11	24.47 ± 2.48	0.69 ± 0.07	15.02 ± 1.13	1.48 ± 0.15	15.18 ± 1.06	0.57 ± 0.05	12.26 ± 0.78
**Gomisin J**	0.24 ± 0.01	7.17 ± 0.79	0.44 ± 0.04	11.58 ± 1.18	0.29 ± 0.03	8.84 ± 0.66	0.60 ± 0.06	8.18 ± 0.57	0.22 ± 0.02	5.79 ± 0.37
**Gomisin A**	3.00 ± 0.09	51.10 ± 5.65	3.84 ± 0.38	81.59 ± 8.28	2.92 ± 0.31	56.64 ± 4.25	5.62 ± 0.59	53.51 ± 3.74	1.77 ± 0.15	45.03 ± 2.84
**Gomisin G**	3.33 ± 0.10	3.39 ± 0.38	3.42 ± 0.34	5.94 ± 0.60	1.95 ± 0.21	3.75 ± 0.28	3.13 ± 0.33	3.81 ± 0.27	2.71 ± 0.22	2.66 ± 0.17
**Licarin B**	0.10 ± 0.003	0.07 ± 0.01	0.14 ± 0.01	0.14 ± 0.01	0.05 ± 0.01	traces	0.11 ± 0.01	0.15 ± 0.01	0.09 ± 0.01	0.13 ± 0.01
**Epigomisin O**	0.43 ± 0.01	0.13 ± 0.01	0.59 ± 0.06	0.24 ± 0.03	0.23 ± 0.03	0.14 ± 0.01	0.57 ± 0.06	0.17 ± 0.01	0.33 ± 0.03	0.12 ± 0.01
**Gomisin O**	2.59 ± 0.08	0.63 ± 0.1	3.10 ± 0.31	1.43 ± 0.15	1.42 ± 0.15	0.76 ± 0.06	2.78 ± 0.30	0.88 ± 0.06	1.84 ± 0.15	0.61 ± 0.04
**Meso-dihydroguaiaretic acid**	0.07 ± 0.002	0.06 ± 0.01	0.09 ± 0.01	0.09 ± 0.01	traces	0.09 ± 0.01	0.08 ± 0.01	0.10 ± 0.01	0.07 ± 0.01	0.07 ± 0.01
**Schisantherin A**	2.38 ± 0.07	0.16 ± 0.02	2.29 ± 0.23	0.33 ± 0.03	1.43 ± 0.15	0.16 ± 0.01	2.33 ± 0.24	0.35 ± 0.03	1.38 ± 0.11	0.19 ± 0.01
**Schisantherin B**	1.32 ± 0.04	6.69 ± 0.74	1.54 ± 0.15	12.57 ± 1.28	0.88 ± 0.09	7.50 ± 0.56	1.85 ± 0.19	10.04 ± 0.70	1.00 ± 0.08	7.45 ± 0.47
**Licarin A**	12.30 ± 0.38	5.81 ± 0.64	14.85 ± 1.48	10.50 ± 1.07	20.41 ± 2.15	10.12 ± 0.76	34.71 ± 3.62	11.52 ± 0.81	31.81 ± 2.60	8.97 ± 0.56
**Schisanhenol**	6.06 ± 0.19	2.19 ± 0.24	5.74 ± 0.57	4.14 ± 0.42	3.32 ± 0.35	2.82 ± 0.21	6.94 ± 0.72	3.43 ± 0.24	4.79 ± 0.39	2.48 ± 0.16
**Deoxyschisandrin**	8.69 ± 0.27	53.87 ± 5.96	9.05 ± 0.90	82.87 ± 8.41	6.61 ± 0.70	58.91 ± 4.42	14.50 ± 1.51	77.72 ± 5.44	7.39 ± 0.60	59.76 ± 3.76
**Fragransin A**	traces	traces	traces	traces	traces	traces	traces	traces	traces	traces
**Pregomisin**	traces	traces	traces	traces	traces	traces	traces	traces	traces	traces
**Gomisin N**	2.15 ± 0.07	13.88 ± 1.53	2.26 ± 0.23	20.49 ± 2.08	1.53 ± 0.16	13.65 ± 1.02	3.08 ± 0.32	18.82 ± 1.32	1.55 ± 0.13	13.85 ± 0.88
**6-O-Benzylgomisin O**	0.42 ± 0.01	0.58 ± 0.06	0.44 ± 0.04	0.79 ± 0.08	0.28 ± 0.03	0.56 ± 0.04	0.49 ± 0.05	0.71 ± 0.05	0.33 ± 0.03	0.49 ± 0.03
**Schisandrin C**	traces	2.98 ± 0.33	0.16 ± 0.02	4.16 ± 0.42	0.12 ± 0.01	2.77 ± 0.21	0.19 ± 0.02	3.17 ± 0.22	0.08 ± 0.01	2.43 ± 0.15
**Total content**	**55.50 ± 1.32**	**194.10 ± 1.46**	**60.30 ± 6.03**	**323.30 ± 32.81**	**48.20 ± 5.09**	**225.20 ± 6.88**	**95.00 ± 9.95**	**254.40 ± 17.81**	**64.55 ± 5.24**	**200.21 ± 12.62**

Table S3. The lignan production (mg/100 g DW±SD) in experimental F and M lines of *S. rubriflora* microshoot cultures after elicitation with yeast extract; traces = <0.05 mg/100 g DW.

Lignans	Time of biomass harvesting after elicitation									
	24h		48h		4 days		6 days		8 days	
	F	M	F	M	F	M	F	M	F	M
**Wulignan A1**	0.05 ± 0.002	traces	traces	traces	0.11 ± 0.01	traces	0.26 ± 0.02	traces	0.31 ± 0.04	traces
**Rubisandrin A**	traces	traces	traces	traces	traces	0.14 ± 0.01	traces	0.19 ± 0.01	traces	0.15 ± 0.01
**Intheriotherin C**	traces	traces	traces	traces	traces	traces	traces	traces	traces	traces
**Schisandrin**	11.09 ± 0.49	60.19 ± 5.65	9.83 ± 1.11	56.37 ± 4.50	10.43 ± 0.63	70.80 ± 5.71	14.74 ± 0.88	57.94 ± 4.13	17.49 ± 2.02	47.87 ± 3.68
**Gomisin D**	1.19 ± 0.05	21.45 ± 2.05	0.36 ± 0.04	20.04 ± 1.60	1.97 ± 0.12	25.86 ± 2.13	2.84 ± 0.17	20.12 ± 1.44	1.66 ± 0.2	17.35 ± 1.33
**Gomisin J**	0.54 ± 0.02	13.67 ± 1.16	0.14 ± 0.02	10.17 ± 0.81	0.78 ± 0.05	14.11 ± 1.19	1.47 ± 0.09	12.37 ± 0.88	0.63 ± 0.08	8.90 ± 0.69
**Gomisin A**	6.03 ± 0.27	77.84 ± 7.38	1.38 ± 0.16	66.50 ± 5.31	8.05 ± 0.49	85.79 ± 6.96	11.32 ± 0.68	68.37 ± 4.87	5.41 ± 0.65	58.65 ± 4.51
**Gomisin G**	2.65 ± 0.12	6.19 ± 0.57	3.06 ± 0.34	4.26 ± 0.34	2.59 ± 0.16	6.53 ± 0.55	3.06 ± 0.18	4.74 ± 0.34	4.08 ± 0.49	4.25 ± 0.33
**Licarin B**	0.12 ± 0.01	0.07 ± 0.01	0.15 ± 0.02	0.14 ± 0.01	0.07 ± 0.004	0.15 ± 0.01	traces	0.06 ± 0.004	0.16 ± 0.02	0.07 ± 0.01
**Epigomisin O**	0.35 ± 0.02	0.27 ± 0.03	0.43 ± 0.05	0.22 ± 0.02	0.28 ± 0.02	0.27 ± 0.02	0.36 ± 0.02	0.20 ± 0.01	0.69 ± 0.08	0.19 ± 0.02
**Gomisin O**	2.32 ± 0.10	1.44 ± 0.13	2.82 ± 0.32	1.23 ± 0.10	1.58 ± 0.10	1.47 ± 0.12	2.301 ± 0.14	1.10 ± 0.08	3.36 ± 0.41	0.83 ± 0.06
**Meso-dihydroguaiaretic acid**	traces	0.14 ± 0.01	0.05 ± 0.01	0.11 ± 0.01	0.06 ± 0.003	0.15 ± 0.01	0.07 ± 0.004	0.11 ± 0.01	0.09 ± 0.01	0.07 ± 0.01
**Schisantherin A**	2.07 ± 0.09	0.53 ± 0.04	2.25 ± 0.25	0.39 ± 0.03	1.78 ± 0.11	0.47 ± 0.04	2.21 ± 0.13	0.31 ± 0.02	2.49 ± 0.27	0.22 ± 0.02
**Schisantherin B**	1.51 ± 0.07	11.81 ± 1.10	1.21 ± 0.14	10.65 ± 0.85	1.61 ± 0.10	13.34 ± 1.10	2.12 ± 0.13	10.69 ± 0.76	2.18 ± 0.26	9.02 ± 0.69
**Licarin A**	5.19 ± 0.23	3.54 ± 0.34	7.48 ± 0.84	3.58 ± 0.29	16.32 ± 0.99	3.46 ± 0.26	35.38 ± 2.11	3.27 ± 0.23	37.54 ± 4.51	2.68 ± 0.21
**Schisanhenol**	4.03 ± 0.18	4.02 ±0.38	5.60 ± 0.63	4.02 ± 0.32	3.94 ± 0.24	4.67 ± 0.38	5.53 ± 0.33	4.08 ± 0.29	7.52 ± 0.88	3.31 ± 0.25
**Deoxyschisandrin**	10.25 ± 0.45	85.16 ± 7.91	7.40 ± 0.83	77.42 ± 6.18	11.45 ± 0.70	94.86 ± 7.56	17.40 ± 1.04	80.44 ± 5.73	16.14 ± 1.90	69.68 ± 5.34
**Fragransin A**	traces	traces	traces	traces	traces	traces	traces	traces	traces	traces
**Pregomisin**	traces	traces	traces	traces	traces	traces	traces	traces	traces	traces
**Gomisin N**	2.56 ± 0.11	23.79 ± 2.14	1.86 ± 0.21	18.56 ± 1.48	2.46 ± 0.15	24.89 ± 2.05	3.88 ± 0.23	20.49 ± 1.46	3.67 ± 0.43	17.52 ± 1.34
**6-O-Benzylgomisin O**	0.41 ± 0.02	1.21 ± 0.11	0.44 ± 0.05	0.89 ± 0.07	0.35 ± 0.02	1.12 ± 0.09	0.48 ± 0.03	1.12 ± 0.08	0.64 ± 0.08	0.85 ± 0.07
**Schisandrin C**	0.19 ± 0.01	5.24 ± 0.47	traces	3.58 ± 0.29	0.30 ± 0.02	5.05 ± 0.42	0.52 ± 0.03	3.93 ± 0.28	0.24 ± 0.03	3.44 ± 0.27
**Total content**	**50.60 ± 2.22**	**316.65 ± 29.46**	**44.60 ± 5.01**	**278.19 ± 22.22**	**64.10 ± 3.90**	**353.17 ± 28.62**	**103.90 ± 6.20**	**289.57 ± 20.63**	**104.29 ± 12.35**	**245.13 ± 18.83**

Table S4. The lignan production (mg/100 g DW±SD) in experimental F and M lines of *S. rubriflora* microshoot cultures after elicitation with ethephon; traces = <0.05 mg/100 g DW.

**Lignans**	**Time of biomass harvesting after elicitation**									
	**24h**		**48h**		**4 days**		**6 days**		**8 days**	
	**F**	**M**	**F**	**M**	**F**	**M**	**F**	**M**	**F**	**M**
**Wulignan A1**	traces	traces	0.07 ± 0.01	traces	traces	traces	traces	traces	traces	traces
**Rubisandrin A**	traces	traces	traces	traces	traces	traces	traces	traces	traces	traces
**Intheriotherin C**	traces	traces	traces	traces	traces	traces	traces	traces	traces	traces
**Schisandrin**	6.20 ± 0.60	60.64 ± 6.50	29.61 ± 3.92	43.15 ± 3.84	14.90 ± 0.95	57.02 ± 2.45	13.74 ± 0.84	25.62 ± 1.98	14.52 ± 1.05	29.40 ± 2.14
**Gomisin D**	0.16 ± 0.02	20.48 ± 2.20	3.84 ± 0.51	15.51 ± 1.38	0.89 ± 0.06	22.50 ± 0.97	1.49 ± 0.09	7.04 ± 0.54	1.11 ± 0.08	6.91 ± 0.50
**Gomisin J**	traces	10.00 ± 1.07	1.84 ± 0.24	7.66 ± 0.68	0.57 ± 0.04	14.59 ± 0.63	0.64 ± 0.04	2.69 ± 0.21	0.44 ± 0.03	2.97 ± 0.22
**Gomisin A**	0.74 ± 0.07	74.62 ± 8.00	15.90 ± 2.11	54.04 ± 4.81	5.78 ± 0.37	82.32 ± 3.54	7.04 ± 0.42	30.24 ± 2.33	5.86 ± 0.42	37.00 ± 2.69
**Gomisin G**	1.92 ± 0.19	4.94 ± 0.53	6.53 ± 0.87	3.84 ± 0.34	4.93 ± 0.31	6.76 ± 0.29	3.43 ± 0.21	1.50 ± 0.12	4.31 ± 0.31	2.07 ± 0.15
**Licarin B**	0.11 ± 0.01	0.07 ± 0.01	0.41 ± 0.06	0.24 ± 0.02	0.17 ± 0.01	0.10 ± 0.004	0.22 ± 0.01	0.11 ± 0.01	0.15 ± 0.01	0.14 ± 0.01
**Epigomisin O**	0.24 ± 0.02	0.21 ± 0.02	1.54 ± 0.20	0.27 ± 0.02	0.93 ± 0.06	0.26 ± 0.01	0.55 ± 0.03	0.08 ± 0.01	0.54 ± 0.04	0.11 ± 0.01
**Gomisin O**	1.34 ± 0.13	1.16 ± 0.12	4.47 ± 0.59	1.18 ± 0.11	3.73 ± 0.24	1.51 ± 0.07	3.04 ± 0.19	0.38 ± 0.03	2.70 ± 0.19	0.55 ± 0.04
**Meso-dihydroguaiaretic acid**	traces	0.12 ± 0.01	0.16 ± 0.02	0.08 ± 0.01	0.06 ± 0.004	0.13 ± 0.01	0.06 ± 0.004	traces	0.06 ± 0.004	traces
**Schisantherin A**	1.29 ± 0.13	0.32 ± 0.04	5.42 ± 0.72	0.51 ± 0.05	3.10 ± 0.20	0.58 ± 0.03	2.42 ± 0.15	0.10 ± 0.01	3.01 ± 0.22	0.18 ± 0.01
**Schisantherin B**	0.64 ± 0.06	12.74 ± 1.37	4.73 ± 0.63	9.45 ± 0.84	2.16 ± 0.14	15.17 ± 0.65	2.09 ± 0.13	4.23 ± 0.33	1.89 ± 0.14	4.98 ± 0.36
**Licarin A**	2.76 ± 0.27	2.86 ± 0.31	6.81 ± 0.90	2.95 ± 0.26	3.10 ± 0.20	6.14 ± 0.26	5.48 ± 0.33	3.56 ± 0.28	5.39 ± 0.39	2.84 ± 0.21
**Schisanhenol**	2.35 ± 0.23	4.09 ± 0.44	10.11 ± 1.34	3.24 ± 0.29	6.32 ± 0.40	4.43 ± 0.19	5.74 ± 0.35	1.74 ± 0.13	4.44 ± 0.32	2.03 ± 0.15
**Deoxyschisandrin**	3.58 ± 0.35	91.53 ± 9.82	23.55 ± 3.12	73.33 ± 6.53	11.58 ± 0.74	91.58 ± 3.93	14.19 ± 0.86	39.93 ± 3.08	10.53 ± 0.76	49.35 ± 3.60
**Fragransin A**	traces	traces	traces	traces	traces	traces	traces	traces	traces	traces
**Pregomisin**	traces	traces	traces	traces	traces	traces	traces	traces	traces	traces
**Gomisin N**	0.92 ± 0.09	22.00 ± 2.36	7.57 ± 1.00	17.89 ± 1.59	3.48 ± 0.22	25.71 ±0.01	3.21 ± 0.20	7.60 ± 0.59	3.11 ± 0.22	10.33 ± 0.75
**6-O-benzylgomisin O**	0.21 ± 0.02	1.01 ± 0.11	1.40 ± 0.19	0.89 ± 0.08	0.82 ± 0.05	1.21 ± 0.05	0.51 ± 0.03	0.29 ± 0.02	0.54 ± 0.04	0.42 ± 0.03
**Schisandrin C**	traces	3.54 ± 0.38	0.52 ± 0.07	2.86 ± 0.26	0.19 ± 0.01	4.81 ± 0.21	0.24 ± 0.02	1.10 ± 0.09	0.17 ± 0.01	1.47 ± 0.11
**Total content**	**22.60 ± 2.19**	**310.40 ± 33.28**	**124.50 ± 16.50**	**237.10 ± 21.11**	**62.80 ± 4.00**	**334.90 ± 14.39**	**64.10 ± 3.90**	**126.30 ± 9.74**	**58.80 ± 4.23**	**150.80 ± 10.97**

Table S5. The lignan production (mg/100 g DW±SD) in experimental F and M lines of *S. rubriflora* microshoot cultures after elicitation with methyl jasmonate; traces = <0.05 mg/100 g DW.

**Lignans**	**Time of biomass harvesting after elicitation**									
	**24h**		**48h**		**4 days**		**6 days**		**8 days**	
	**F**	**M**	**F**	**M**	**F**	**M**	**F**	**M**	**F**	**M**
**Wulignan A1**	0.07 ± 0.01	traces	0.06 ± 0.003	traces	0.12 ± 0.01	0.06 ± 0.01	0.11 ± 0.01	traces	traces	0.05 ± 0.01
**Rubisandrin A**	traces	traces	traces	traces	traces	traces	traces	traces	traces	traces
**Intheriotherin C**	traces	traces	traces	traces	traces	traces	traces	traces	traces	traces
**Schisandrin**	7.95 ± 0.94	71.98 ± 11.33	22.21 ± 1.24	34.25 ± 3.07	9.14 ± 0.80	35.16 ± 3.30	14.84 ± 1.42	28.92 ± 1.23	10.22 ± 0.92	56.95 ± 5.09
**Gomisin D**	0.23 ± 0.03	28.80 ± 4.53	1.97 ± 0.11	8.30 ± 0.74	0.45 ± 0.04	10.59 ± 0.99	0.59 ± 0.06	11.42 ± 0.49	0.77 ± 0.07	18.53 ± 1.66
**Gomisin J**	0.09 ± 0.01	15.71 ± 2.47	0.73 ± 0.04	5.16 ± 0.46	0.26 ± 0.02	5.00 ± 0.470	0.30 ± 0.03	4.76 ± 0.20	0.35 ± 0.07	9.23 ± 0.83
**Gomisin A**	1.32 ± 0.16	91.53 ± 14.40	7.42 ± 0.42	41.11 ± 3.68	2.59 ± 0.23	44.74 ± 4.20	3.24 ± 0.31	38.78 ± 1.65	3.72 ± 0.33	69.87 ± 6.24
**Gomisin G**	2.56 ± 0.30	6.93 ± 1.09	6.04 ± 0.34	1.92 ± 0.17	2.79 ± 0.25	2.71 ± 0.25	3.97 ± 0.38	3.03 ± 0.13	3.02 ± 0.27	4.52 ± 0.40
**Licarin B**	0.12 ± 0.01	0.12 ± 0.02	0.21 ± 0.01	0.08 ± 0.01	0.09 ± 0.01	0.09 ± 0.01	0.28 ± 0.03	traces	0.19 ± 0.02	0.10 ± 0.01
**Epigomisin O**	0.35 ± 0.04	0.29 ± 0.05	1.00 ± 0.06	0.12 ± 0.01	0.26 ± 0.02	0.12 ± 0.01	0.49 ± 0.05	0.10 ± 0.004	0.39 ± 0.04	0.19 ± 0.02
**Gomisin O**	2.26 ± 0.27	1.82 ± 0.27	4.80 ± 0.27	0.60 ± 0.05	1.34 ± 0.12	0.55 ± 0.05	2.64 ± 0.25	0.62 ± 0.03	2.37 ± 0.21	0.93 ± 0.08
**Meso-dihydroguaiaretic acid**	traces	0.13 ± 0.02	0.11 ± 0.01	0.07 ± 0.01	0.06 ± 0.01	0.07 ± 0.01	0.08 ± 0.01	0.06 ± 0.003	traces	0.23 ± 0.02
**Schisantherin A**	2.05 ± 0.24	0.46 ± 0.07	4.19 ± 0.23	0.18 ± 0.02	1.66 ± 0.15	0.15 ± 0.01	2.77 ± 0.27	0.14 ± 0.01	2.11 ± 0.19	0.42 ± 0.04
**Schisantherin B**	0.80 ± 0.09	16.66 ± 2.62	2.82 ± 0.16	5.35 ± 0.48	0.89 ± 0.08	6.43 ± 0.60	1.57 ± 0.15	5.60 ± 0.24	1.28 ± 0.12	10.21 ± 0.91
**Licarin A**	9.71 ± 1.15	1.81 ± 0.29	8.68 ± 0.49	4.19 ± 0.38	30.83 ± 2.07	3.88 ± 0.36	9.64 ± 0.93	1.80 ± 0.08	5.04 ± 0.45	4.04 ± 0.36
**Schisanhenol**	3.59 ± 0.43	4.74 ± 0.75	7.97 ± 0.45	2.28 ± 0.20	2.94 ± 0.26	2.11 ± 0.20	5.95 ± 0.57	1.80 ± 0.08	5.12 ± 0.46	3.30 ± 0.30
**Deoxyschisandrin**	4.57 ± 0.54	93.04 ± 14.64	15.86 ± 0.89	53.01 ± 4.75	6.49 ± 0.57	55.33 ± 5.19	13.13 ± 1.26	43.25 ± 1.84	10.08 ± 0.91	80.06 ± 7.15
**Fragransin A**	traces	traces	traces	traces	traces	traces	traces	traces	traces	traces
**Pregomisin**	traces	traces	traces	traces	traces	traces	traces	traces	traces	traces
**Gomisin N**	1.68 ± 0.20	28.07 ± 4.42	4.55 ± 0.25	12.04 ± 1.08	1.63 ± 0.14	12.52 ± 1.17	2.90 ± 0.28	10.24 ± 0.44	2.05 ± 0.18	19.55 ± 1.75
**6-O-Benzylgomisin O**	0.32 ± 0.04	1.22 ± 0.19	0.82 ± 0.05	0.40 ± 0.04	0.34 ± 0.03	0.52 ± 0.05	0.57 ± 0.06	0.40 ± 0.02	0.38 ± 0.03	0.83 ± 0.07
**Schisandrin C**	0.06 ± 0.01	5.17 ± 0.81	0.24 ± 0.01	1.88 ± 0.17	0.07 ± 0.01	1.94 ± 0.18	0.10 ± 0.01	1.67 ± 0.07	0.15 ± 0.01	3.08 ± 0.28
**Total content**	**37.80 ± 4.47**	**368.50 ± 57.98**	**89.70 ± 5.01**	**171.00 ± 15.31**	**61.90 ± 4.80**	**182.00 ± 17.07**	**63.20 ± 6.07**	**152.70 ± 6.50**	**47.33 ± 4.26**	**47.30 ± 25.21**
